# Early structural connectivity within the sensorimotor network: Deviations related to prematurity and association to neurodevelopmental outcome

**DOI:** 10.3389/fnins.2022.932386

**Published:** 2022-11-25

**Authors:** Sara Neumane, Andrea Gondova, Yann Leprince, Lucie Hertz-Pannier, Tomoki Arichi, Jessica Dubois

**Affiliations:** ^1^Inserm, NeuroDiderot, Université Paris Cité, Paris, France; ^2^CEA, NeuroSpin UNIACT, Université Paris-Saclay, Paris, France; ^3^School of Biomedical Engineering and Imaging Sciences, Centre for the Developing Brain, King’s College London, London, United Kingdom; ^4^Paediatric Neurosciences, Evelina London Children’s Hospital, Guy’s and St Thomas’ NHS Foundation Trust, London, United Kingdom

**Keywords:** brain development, tractography, diffusion MRI (dMRI), Diffusion Tensor Imaging (DTI), NODDI (neurite orientation dispersion and density imaging), multivariate Mahalanobis distance, preterm at term-equivalent age, white matter microstructure maturation

## Abstract

Consisting of distributed and interconnected structures that interact through cortico-cortical connections and cortico-subcortical loops, the sensorimotor (SM) network undergoes rapid maturation during the perinatal period and is thus particularly vulnerable to preterm birth. However, the impact of prematurity on the development and integrity of the emerging SM connections and their relationship to later motor and global impairments are still poorly understood. In this study we aimed to explore to which extent the early microstructural maturation of SM white matter (WM) connections at term-equivalent age (TEA) is modulated by prematurity and related with neurodevelopmental outcome at 18 months corrected age. We analyzed 118 diffusion MRI datasets from the developing Human Connectome Project (dHCP) database: 59 preterm (PT) low-risk infants scanned near TEA and a control group of full-term (FT) neonates paired for age at MRI and sex. We delineated WM connections between the primary SM cortices (S1, M1 and paracentral region) and subcortical structures using probabilistic tractography, and evaluated their microstructure with diffusion tensor imaging (DTI) and neurite orientation dispersion and density imaging (NODDI) models. To go beyond tract-specific univariate analyses, we computed a *maturational distance related to prematurity* based on the multi-parametric Mahalanobis distance of each PT infant relative to the FT group. Our results confirmed the presence of microstructural differences in SM tracts between PT and FT infants, with effects increasing with lower gestational age at birth. Maturational distance analyses highlighted that prematurity has a differential effect on SM tracts with higher distances and thus impact on (i) cortico-cortical than cortico-subcortical connections; (ii) projections involving S1 than M1 and paracentral region; and (iii) the most rostral cortico-subcortical tracts, involving the lenticular nucleus. These different alterations at TEA suggested that vulnerability follows a specific pattern coherent with the established WM caudo-rostral progression of maturation. Finally, we highlighted some relationships between NODDI-derived maturational distances of specific tracts and fine motor and cognitive outcomes at 18 months. As a whole, our results expand understanding of the significant impact of premature birth and early alterations on the emerging SM network even in low-risk infants, with possible relationship with neurodevelopmental outcomes. This encourages further exploration of these potential neuroimaging markers for prediction of neurodevelopmental disorders, with special interest for subtle neuromotor impairments frequently observed in preterm-born children.

## Introduction

The cerebral somatosensory and motor systems consist of distributed networks of specialized interconnected cortical and subcortical gray matter (GM) regions, interacting through white matter (WM) tracts, that support a wide variety of sensory and motor functions that are essential for nearly every human behavior across the lifespan. In somatosensation, inputs from peripheral receptors are first conveyed by peripheral nerves, then through the spinal cord to the *brainstem* dorsal column nuclei. These nuclei further connect to the thalamus which sends projections to cortical somatosensory areas, particularly the *primary somatosensory cortex* (S1) located on the *postcentral gyrus*. On the other hand, the *primary motor cortex* (M1) in the *precentral gyrus*, is critical for motor behavior, exerting its influence over the body’s muscles through its output to a variety of descending pathways, the main being the direct cortical innervation of motoneurons *via* the corticospinal tract (CST). S1 and M1 are reciprocally connected, *directly via* short-range intra-hemispheric and homotopic interhemispheric pathways, and *indirectly via* some cortico-subcortical pathways predominately involving the *thalamus* and the *basal ganglia* (BG). The BG notably include the *caudate nucleus*, *putamen* and *globus pallidus*: the first two functionally constitute the striatum (receiving most of the BG inputs), while the last two are grouped anatomically in the lenticular nucleus, with the globus pallidus representing one of the key output structures of the BG (Leisman et al., 2014).

Interactions between somatosensory and motor systems, observable in mature brains (Hatsopoulos and Suminski, 2011; Tomasino and Gremese, 2016), are particularly important during the early stages of neurodevelopment. The late second and third trimesters of gestation, as well as the neonatal period, are a critical time for the dynamic refinement and maturation of brain networks through several complex processes (Dubois et al., 2014; Kostović et al., 2019), laying the foundations of structural connectivity that underlie later neurodevelopment (Gilmore et al., 2018). As projection and interhemispheric tracts show rapid growth before 28 weeks of gestational age (wGA) (Keunen et al., 2017; Kostović et al., 2019), the general architecture of the *sensorimotor* (SM) network is already established during the preterm period, making it one of the earliest brain systems to mature (Dubois et al., 2014; Ouyang et al., 2019; Machado-Rivas et al., 2021). It may therefore play a pivotal role for the optimal development of secondary and associative networks in their earliest stages and for organizing the structural and functional connectome throughout the neonatal period (Ball et al., 2014; van den Heuvel et al., 2015; Zhao et al., 2019).

This crucial maturation phase is also highlighted by the adverse effects of preterm birth (before 37wGA) on neurodevelopment. The sudden need to adapt to extra-uterine life does not provide the optimal conditions for physiological neurodevelopmental mechanisms, resulting in variable structural and/or functional abnormalities (Suzuki, 2007). The related diffuse cerebral dysmaturation (Back, 2015; Volpe, 2021) alters the integrity of the emerging neural networks (Suzuki, 2007; Back, 2015), with early maturing regions suffering the largest adverse effects with a greater degree of prematurity (lower GA at birth) (Knight et al., 2018).

Magnetic resonance imaging (MRI) including diffusion MRI (dMRI) has been extensively used to evaluate the consequences of preterm birth on brain development. Even in the absence of focal cerebral lesions, prematurity is associated with disturbances in brain growth, in particular in GM structures including the BG and thalamus (Keunen et al., 2012; Padilla et al., 2015; Loh et al., 2020), and pervasive widespread abnormalities in GM and WM microstructure, maturation and connectivity (Ball et al., 2013b; Batalle et al., 2018). In particular, the WM of preterm infants at term-equivalent age (TEA) has a more “immature” microstructural profile compared with term-born neonates, consistent with delayed and/or disrupted WM development and maturation (Thompson et al., 2011; Kelly et al., 2016a,^2020^). The extent of early WM abnormalities (even in the absence of overt brain lesion) has been related to poorer neurodevelopmental outcome (Duerden et al., 2015; Barnett et al., 2018; Kelly et al., 2020; Pannek et al., 2020). Preterm infants are also at higher risk of impaired neuromotor function (Williams et al., 2010; Odd et al., 2013; Spittle and Orton, 2014), that can manifest as poorer fine and gross motor skills compared with term-born controls (Evensen et al., 2020). Although long-lasting WM alterations in SM tracts have been observed during childhood and adolescence in these populations (Groeschel et al., 2014; Dewey et al., 2019; Thompson et al., 2020), the relationship between neonatal SM network structural alterations and neuromotor impairment in *low-risk preterm* infants (including moderate to late preterm and/or preterm babies without perinatal brain injury) has been less systematically explored. Also, assessing early WM *maturational delays* across the different SM tracts and analyzing the correlation with outcome would enable a better understanding of the pathophysiology of the disorders resulting from deviations in typical developmental processes.

In this study, we thus aimed to assess how preterm birth impacts SM network maturation at TEA in the absence of overt cerebral lesions, and the potential effect on later neurodevelopmental outcome. We hypothesized that SM network would show significantly altered microstructure in preterm infants compared to full-term neonates, with distinct patterns of maturation delay across the SM tracts, and that these alterations are associated with motor and global neurodevelopmental outcomes. For this purpose, we studied a large cohort of low-risk preterm infants at TEA and full-term neonates from the *developing Human Connectome Project* (dHCP) (Edwards et al., 2022), and investigated the effects of prematurity on WM microstructure and maturation of SM tracts using complementary approaches based on diffusion MRI data and tractography (Dubois et al., 2014, 2016; Ouyang et al., 2019). We dissected an unprecedented set of SM cortico-cortical and cortico-subcortical tracts and computed quantitative metrics from two complementary models: the widely used Diffusion Tensor Imaging (DTI) model (Dubois et al., 2014; Pecheva et al., 2018; Ouyang et al., 2019) and the more specific 3 tissue compartments model of neurite orientation dispersion and density imaging (NODDI) (Zhang et al., 2012; Kunz et al., 2014; Batalle et al., 2017; Kimpton et al., 2021). To overcome the limitations inherent to univariate dMRI approaches which cannot reflect the inter-related complexity of processes involved in early brain maturation, we used the *multivariate Mahalanobis distance* approach to compare the preterm and full-term groups (Kulikova et al., 2015; Dean et al., 2017; Li et al., 2022). This allowed us to consider multiple metrics and to quantify the tract-specific maturational gap at TEA between a preterm infant and the reference group of full-term neonates, with the added advantage of taking into account inter-subject variability in the reference group, as well as correlations between input metrics. Moreover, using the Mahalonobis distance measure with different sets of DTI and NODDI complementary metrics allowed the effect of maturation and complex underlying WM microstructural processes to be accounted for. Finally, we evaluated the relationships between the aforementioned distances for the different SM connections and neurodevelopmental outcome at 18 months of corrected age (mCA).

## Materials and methods

### Subjects

This study included a sample of preterm and full-term neonates taken from the dHCP cohort, collected at St Thomas’ Hospital London, UK from 2015 to 2020.^1^ This project received UK NHS research ethics committee approval (14/LO/1169, IRAS 138070), and written informed consent was obtained from the parents of all participant infants. From the overall cohort, we identified 59 preterm (PT) infants (33 males, gestational age at birth – GA at birth: median 31.7 weeks, range [23.7w–36.0w]) scanned near TEA (median post-menstrual age –PMA: 41.3w, range [38.4w–44.9w]), and a control group of 59 full-term born (FT) infants (GA at birth: median 40.1w, range [37.4w–42.3w]) matched to the preterm population on age at MRI and sex. Preterm infants were subdivided into infants born extremely to very preterm (GA at birth < 32w, *N* = 33; PT_EV_ group) or moderate to late preterm (GA at birth ≥ 32w, *N* = 26; PT_ML_ group). The corresponding controls are subsequently noted FT_EVCt_ and FT_MLCt_, respectively. All included infants were deemed healthy at TEA, i.e., were without major brain focal lesions or any overt abnormality of clinical significance on structural MRI as evaluated by an expert pediatric neuroradiologist (dHCP radiological score in the range 1*--*3).^2^

#### Neonatal characteristics at birth

*Obstetric factors* (i.e., multiple pregnancy status, intrauterine growth restriction –IUGR, maternal antenatal steroids and magnesium therapy, delivery method) as well as *infant characteristics at birth* (i.e., Apgar scores at 1 and 5 min, birth weight, length, and head circumference) were extracted from the dHCP records.

Specific *postnatal risk factors* previously recognized to be related with neonatal brain abnormalities, including diffuse and regional WM microstructural alterations (Pogribna et al., 2013; Brouwer et al., 2017; Barnett et al., 2018; Parikh et al., 2021), were also considered. These included NICU variables (i.e., total duration of ventilatory support and oxygen therapy, and parenteral nutrition) which were binarized using thresholds established in previous studies (need of mechanical ventilation beyond 7 days, and of parenteral nutrition longer than 21 days) (Brouwer et al., 2017). Sepsis was considered as any situation where an infant received antibiotics, as there was not enough information to retain only confirmed episodes of postnatal sepsis. Additionally, we derived a *neonatal morbidities* binary factor to summarize the presence of at least one of the following 4 morbidities associated with prematurity (or the absence of all 4): chronic lung disease, necrotizing enterocolitis (NEC), retinopathy of prematurity (ROP) and abnormal cranial ultrasonography (cUS). Of note, detailed neonatal medical records were available only for infants admitted to the neonatal intensive care unit (NICU) after birth: 51 PT (86%) and 2 FT (3%, admitted for sepsis treatment, without further complications).

Comparisons between PT and FT groups in terms of the described variables and factors were performed with suitable tests (Wilcoxon rank sum test for ordinal and continuous variables; Fisher’s exact test for binary factors; Pearson’s Chi-squared test for non-binary nominal factors) in R (version 4.0.5, 2021.03.31).

#### Outcome assessment and infant characteristics at 18 months

*Family socio-economic status* (SES) was measured using the index of multiple deprivation (IMD) which is a UK geographically defined composite social risk score comprising data on income, employment, health, education, living environment, and crime calculated from the mother’s home address at the time of birth.

*Neurodevelopmental outcome* was assessed at St Thomas’ Hospital, London by two experienced assessors using the Bayley Scales of Infant and Toddler Development, Third Edition –BSID-III (Bayley, 2006). We only considered assessments performed at around 18 mCA (between 17 and 21 m), which was available for 44 (75%) PT infants and 53 (90%) FT infants (median age: 18.3 m). Five distinct developmental categories (cognition, receptive and expressive language, and fine and gross motor function) were assessed yielding age-standardized respective *scaled scores* (mean 10, SD 3), with higher values indicating better infant development and scores lower than 7 indicating developmental delay in that domain.

Comparisons between PT and the FT groups were performed with *t*-tests corrected for multiple comparisons using Benjamini–Hochberg false discovery rate (FDR) correction across scores. The effect of sub-groups (PT_EV,_ PT_ML_, FT_EVCt_, and FT_MLCt_) on neurodevelopmental outcomes was assessed using one-way ANOVA.

Of note, the 5 BSID-III scaled scores can be summarized into the widely used 3 *composite* cognitive, language, and motor scores (mean 100, SD 15). The results of the entire analyses performed using them can be found in the Supplementary materials: *BSID-III composite score results* section.

### Magnetic resonance imaging data acquired at term-equivalent age

Magnetic resonance imaging (MRI) data was acquired using a Philips 3-Tesla Achieva scanner (Philips Medical Systems, Best, Netherlands). All infants were scanned during natural sleep using a neonatal head coil and imaging system optimized for the dHCP study as previously described (Hughes et al., 2017).

We used anatomical and diffusion MRI data available in its pre-processed state from the dHCP database (third release) (Edwards et al., 2022). The *structural* data was a result of acquisition and reconstruction using optimized protocols (Cordero-Grande et al., 2018) leading to super-resolved T2w images with an isotropic spatial voxel size of 0.5 mm. Processing followed a dedicated pipeline for segmentation and cortical surface extraction for T2w images of neonatal brains (Makropoulos et al., 2018), with bias-correction, brain extraction, and segmentation using Draw-EM (Developing brain Region Annotation with Expectation Maximisation) algorithm (Makropoulos et al., 2014). White matter surface (inner cortical surface) meshes provided within the dHCP database were used for the segmentation of cortical regions of interest (ROIs), while volumetric segmentations were directly used to extract subcortical ROIs (cf. section *Selection and delineation of regions of interest*). Additionally, derived hemispheric, WM and cortical masks (also referred to as cortical ribbons) were also used for the tractography analysis (cf. section *SM connectivity reconstruction*).

Acquisition and reconstruction of the *diffusion* data followed a multi-shell high angular resolution diffusion imaging (HARDI) protocol with 4 b-shells (*b* = 0 s/mm^2^: 20 repeats; and *b* = 400, 1,000, 2,600 s/mm^2^: 64, 88, and 128 directions, respectively) (Hutter et al., 2018) and was pre-processed with correction for motion artifacts and slice-to-volume reconstruction using the SHARD approach, leading to an isotropic voxel size of 1.5 mm (Christiaens et al., 2021). Pre-processed data was used for the fitting of diffusion models (cf. section *Estimation of diffusion models*) and for the tractography analysis (cf. section *SM connectivity reconstruction*).

### Assessment of sensorimotor network microstructure

To estimate WM microstructural characteristics within the SM network, we first quantified complementary diffusion metrics from the available diffusion data. Structural connections between pairs of anatomically defined SM regions, including cortical primary SM cortices and key sub-cortical structures, were delineated using probabilistic tractography. The diffusion metrics were then extracted for the selected connections of interest and used to study developmental differences between the cohort subgroups.

#### Estimation of diffusion models

The DTI model was fitted to the diffusion data using a single shell (*b* = 1,000 s/mm^2^) and calculated with FSL’s DTIFIT. The choice of using only a single *b*-value was made because the utility of including more diffusion directions may be outweighed by the non-Gaussian contribution of high b-value acquisitions (Pines et al., 2020). DTI maps were computed for four metrics: fractional anisotropy (FA) and Mean Diffusivity (MD) which are a composite of axial diffusivity (AD) and radial diffusivity (RD).

Additionally, multi-shell diffusion data were used to derive the NDI and ODI maps from the NODDI model (Zhang et al., 2012) using the CUDA 9.1 Diffusion Modelling Toolbox (cuDIMOT) NODDI Watson model implementation for GPUs (Hernandez-Fernandez et al., 2019). We used the MCMC optimization algorithm and default settings to fit the NODDI model to our infant data. The NODDI-derived maps were then post-processed to reduce the observed noise. Briefly, we used ODI maps to detect possible errors using an alpha-trimming strategy. The voxels presenting values outside the threshold range (fixed upper value of 0.95 and the lower limit being the first groove of the histogram of values) were either (i) normalized by the immediate surrounding values (i.e., the mean of the voxel’s immediate environment after the removal of extreme values), or (ii) set to 0, if no voxels in the “normal” range were found in their environment. The same erroneous voxels were also corrected in NDI maps in the same fashion.

#### Selection and delineation of regions of interest

**FIGURE 1:**
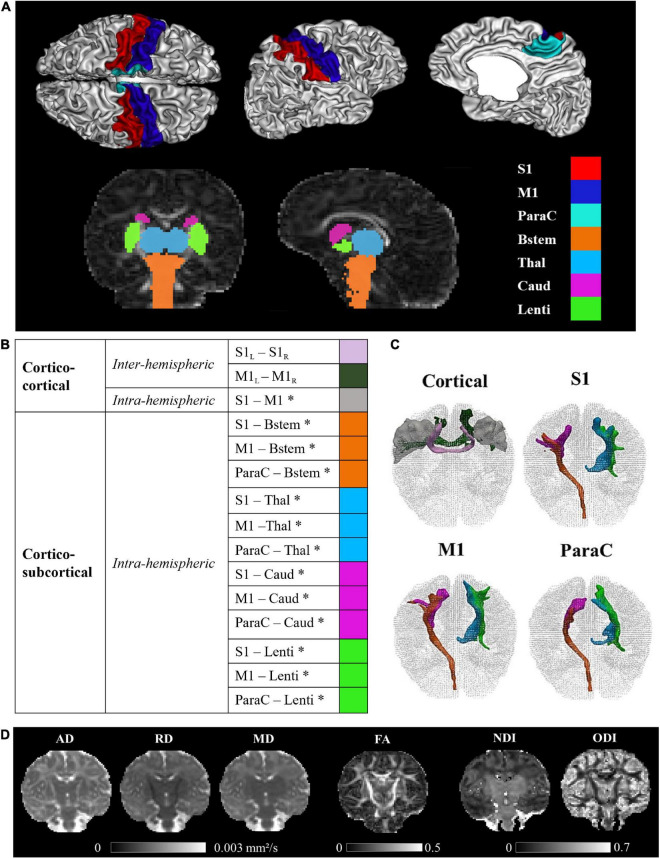
Regions of interest, sensorimotor tracts, and diffusivity metrics for a representative full-term infant (GA at birth 40.4w, PMA at MRI 44.1w). **(A)** Visualization of the cortical and subcortical ROIs used as tractography seeds. **(B)** List of the SM tracts of interest. **(C)** 3D reconstructions of SM tracts. **(D)** Metric maps resulting from DTI (AD, RD, MD, FA) and NODDI (NDI, ODI) models. GA, gestational age (in weeks); PMA, post-menstrual age (in weeks); ROIs, regions of interest; SM, sensorimotor; S1, lateral portion of the primary somatosensory cortex (postcentral gyrus); M1, lateral portion of the primary motor cortex (precentral gyrus); ParaC, medial portions of the primary sensorimotor cortices (paracentral area); Bstem, brainstem; Thal, thalamus; Caud, caudate nucleus; Lenti, lenticular nucleus; L, left; R, right. *Intra-hemispheric tracts, evaluated in left and right hemispheres separately. DTI, diffusion tensor imaging; metrics: AD, axial diffusivity; RD, radial diffusivity; MD, mean diffusivity; FA, fractional anisotropy; NODDI, neurite orientation dispersion and density imagingmetrics; NDI, neurite density index; ODI, orientation dispersion index.

Pre-processed structural data was used to parcellate 13 ROIs (6 in each hemisphere and 1 bilateral, Figure 1A) relevant for the developing SM network, focusing on the primary core of cortical and deep GM structures. Regarding cortical regions, we considered *primary sensory* and *motor cortices* which are essential for processing peripheral somatosensory inputs and for initiating and controlling motor behaviors, through cortico-subcortical loops including *thalamus*, *BG* and *brainstem*. We then disregarded non-primary cortical areas, which mature later during typical development. Three cortical ROIs were thus defined on the cortical surface of each hemisphere using the M-CRIB-S surface-based parcellation tool optimized for the term-born neonates (Adamson et al., 2020) whose labeling scheme replicates the Desikan-Killiany-Tourville (DKT) atlas (Klein and Tourville, 2012): the postcentral gyrus as the anatomical proxy of the lateral portion of the primary somatosensory cortex (hereafter referred to as *S1* for the sake of simplification), the precentral gyrus as the lateral portion of the primary motor cortex (referred to as *M1*), and the paracentral lobule (referred to as *ParaC*) corresponding to the medial surface of the hemisphere in the continuation of the precentral and postcentral gyri, including the medial portions of the primary SM cortices. The central sulcus was required as a landmark to delineate these pre- and post-central regions, explaining the choice of a surfacic parcellation tool. Individual surface ROIs were then projected to the cortical ribbon defined in the anatomical volumes, and further dilated by one voxel into the WM to ease the tractography process.

Regarding sub-cortical structures, the most relevant to be studied at early developmental stages are the main input/output and relay structures, implicating in particular the brainstem, the thalamus, the dorsal striatum (composed of the putamen and caudate nucleus) that can be considered as the main BG input structure for SM projections, and the internal segment of the globus pallidus (GPi), one of the major output structures of the BG (Leisman et al., 2014). Identifying these specific GM structures on MR images is quite challenging in infants due to the inter-subject variability and rapid changes in morphological characteristics and sizes during the perinatal period. In addition, the precise segmentation of structures of interest also depends on the possibilities offered by the tool validated for neonatal population. The subcortical ROIs were thus defined using a volumetric GM parcellation based on Draw-EM algorithm segmentation (Makropoulos et al., 2014) provided within the dHCP data release, namely medial brainstem (*Bstem*) and for each hemisphere: thalamus (*Thal*, fusing high and low intensity regions), caudate nucleus (*Caud*, part of the striatum) and lenticular nucleus (*Lenti*, containing the putamen as well as the GPi).

These cortical ROIs (bilateral M1, S1, and ParaC region) and subcortical ROIs (brainstem and bilateral thalamus, caudate and lenticular nuclei), used as seeds for the tractography, were aligned to the diffusion space with FSL 6.0’s FLIRT.

#### Sensorimotor connectivity reconstruction

Individual dissections of SM connections, which to our knowledge have never been achieved in neonates and infants until now, were performed using an automated tractography-based approach benefitting from multi-shell MRI data. For each subject, probabilistic tractography estimating multiple diffusion orientations within a voxel (Behrens et al., 2007) was used to reconstruct connections between the selected ROI pairs (designated as *tracts* thereafter). Briefly, for each subject, we first modeled crossing fibers within each voxel of the multi-shell diffusion data using a GPU accelerated version of FSL’s Bayesian Estimation of Diffusion Parameters Obtained using Sampling Techniques modelling Crossing Fibres (BEDPOSTX), with default settings apart from the deconvolution model with zeppelins (Hernández et al., 2013). Then, the pre-selected ROIs were used as seed masks to derive region-to-region structural connections using the GPU implementation of the Probabilistic Tractography with crossing fibers (ProbTrackX) available with FSL 6.0 (Hernandez-Fernandez et al., 2019), and the default (one-way) setting with a loop check. The resulting output describe the density of WM connections between the ROI pair.

To improve the tractography results, and to reduce the incidence of erroneous streamlines, we employed exclusion masks. These exclusion masks were based on a mask of CSF created by thresholding the MD maps (voxels with MD > 2.10^–3^mm^2^/s were considered as CSF) and corrected by removing voxels with FA > 0.25 (which might correspond to WM voxels in the corpus callosum but close to the ventricles with CSF partial volume effects). The exclusion masks were further adapted to exclude all other brain structures apart from the considered ROIs pair. Additionally, where the pair of ROIs were ipsilateral, i.e., in a single hemisphere, the entire contralateral hemisphere was also excluded. No supplementary constraints were included in the tractography runs.

Reconstructed tracts were then thresholded at 5% of the maximum fiber density of the evaluated tract. This was not performed for cortico-cortical inter-hemispheric tracts, whose reconstructions were used in their original state due to low streamline numbers.

The final list of SM tracts of interest (corresponding to homotopic inter-hemispheric tracts, short-range S1–M1 intra-hemispheric tracts, and long-range intra-hemispheric cortico-subcortical tracts) is described in Figure 1B We visually validated the accuracy of the tracts reconstructions for several subjects and observed expected topographies (e.g., the S1 and M1 projections toward the ventral anterior and lateral portions of the thalami). Note that the (inter- and intra-hemispheric) cortico-cortical tracts involving paracentral regions could not be evaluated due to frequent and variable tractography errors identified upon visual examination. Also, connections between subcortical structures were not studied because of their proximity which could alter the tractography performance.

#### Extraction of tract-specific metrics

DTI and NODDI-derived metrics (FA, MD, AD, RD, NDI, ODI) were extracted from each individual tract by calculating the weighted average value (metric $\bar{X}$) using the following equation:


$$\bar{X}=\frac{\sum \left(d_{i}\times X_{i}\right)}{\sum d_{i}}$$

where *i* denotes the tract voxels, *d*_*i*_ is the fiber density at voxel *i* of a tract, and *X*_*i*_ is value of the metric at voxel *i* (Hua et al., 2008). This weighted approach gave more weight to the central portion (with higher fiber density) compared to the tract periphery, rendered the measures independent on the number of streamlines assessed by the tractography algorithm, and limited the effect of potential artifacts related to tractography reconstruction.

### Univariate tract-specific analyses

To investigate tract-specific relationships between the diffusion metrics and subject characteristics (prematurity, clinical factors, etc.), we performed three sets of univariate analyses on the tract diffusion metrics (see Supplementary materials: *Descriptive univariate analysis* section for the methods and results). This allowed us to identify parameters for the later multivariate analysis. In the univariate analyses, we did not observe interaction between hemisphere and the infant group for any of the six evaluated metrics justifying the *averaging* of the diffusion metrics over the *left and right tracts*. Additionally, evaluated clinical variables were not associated, or were only weakly associated, with the diffusion metrics in the PT group, which led us not to consider them as confounders. In contrast, we observed a strong association between the tract-specific diffusion metrics and (i) infant group (PT_EV_, PT_ML_, FT) or GA at birth; (ii) PMA at scan; and (iii) WM residuals (estimated as the residuals of the linear model considering the metric averaged over the whole WM mask as a function of GA at birth and PMA at scan). The tract-specific diffusion metrics were therefore adjusted for PMA at scan and WM residuals in all the subsequent analyses that aimed to study the effect of prematurity level or GA at birth. As a proxy of the maturational gap between PT and FT, we calculated the relative percentage difference in the metric values between each PT infant and its matched FT control, and averaged this over the PT_EV_ and PT_ML_ groups independently.

### Multivariate tract-specific analyses on effects of prematurity

In order to characterize the potential difference at TEA between the microstructural profiles of PT infants compared to FT neonates for each tract, we used a previously proposed multiparametric approach based on the *Mahalanobis distance* (Kulikova et al., 2015). The goal was to evaluate the distance between each individual PT infant and the FT group as a reference, by taking into account the inter-subject variability within the FT group and the collinearity between a set of diffusion metrics.

Firstly, we scaled each diffusion metric between [0; 1], considering all tracts and the mean WM in all the PT and FT infants. The tract scaled metrics were then corrected for GA at birth, PMA at scan and WM residuals, considering each of the three groups independently (PT_EV_, PT_ML_, and entire FT group) and keeping the respective group value means. Next, we divided the PT and FT *individual* tract metric values by their respective metric means from the FT group.

For the calculation of the Mahalanobis distance, it is beneficial to choose independent metrics that provide complementary information. With this in mind, we decided to subset the six metrics into three parallel analysis streams based on the nature of the metrics and models used to derive them. AD and RD, which are direct measures of the diffusivity within the tracts, were retained as *set 1*. More complex but commonly used DTI metrics: MD and FA, formed *set 2*. Finally, NODDI metrics (NDI and ODI) formed an independent *set 3* to dissociate them from the more widely established DTI metrics and test their relevance for microstructure in the context of SM network and prematurity.

For a given tract, the Mahalanobis distance (Dtract) for a given PT individual was then computed using the following equation:


$$Dtract\,(\vec{x})=\sqrt{{\left(\vec{x}-\vec{\mu }\right)}^{T}S^{-1}(\vec{x}-\vec{\mu })}$$

where $\vec{x}$ is a multivariate vector describing the PT individual tract-specific metrics, $\vec{\mu }$ = [1, …, 1] is the mean vector for the corresponding FT group, and S is a covariation matrix for diffusion metrics in FT infants.

In the interpretation, the smaller the distance, the closer the individual preterm infant is to the distribution within the control FT cohort. Differences in distances across tracts can thus be interpreted as a differential, tract-specific effect of prematurity on maturation.

Regarding statistical analyses, we first evaluated whether the distances for each of the two PT subgroups were significantly different from 0 (meaning that the PT subgroups are different from the FT reference group) using one-sample Wilcoxon signed rank tests corrected for multiple comparisons (FDR) across all tracts and metric sets. We further evaluated the effect of tracts and PT subgroups on the Mahalanobis distances using global ANOVA modeling with these two factors. We additionally compared distances between the two PT subgroups based on unpaired *t*-tests with FDR correction for multiple comparisons. Both ANOVA and *t*-tests were performed after checking for normality of the Mahalanobis tract values using Shapiro–Wilk test corrected for multiple comparisons across sets.

To establish whether SM tracts were differentially affected by the prematurity level, we compared all possible pairs of tracts within each PT group, using paired *t*-tests corrected for multiple comparisons (FDR) across all studied metric sets and tracts.

We finally evaluated the relationship between this maturational distance related to prematurity and neurodevelopmental outcome. For each tract, we evaluated Pearson’s correlations between the Mahalanobis distances in each metrics set and the 5 BSID-III scaled scores, considering infants with outcome data in each group separately (PT_EV_ *N* = 24; PT_ML_ *N* = 20) given the between-subgroup differences observed in distances but not BSID-III scores (see Results section). The reported results were corrected for multiple comparisons (FDR) across all tract and metric sets.

Statistical analyses were performed in R (version 4.0.5, 2021.03.31). Statistical tests throughout the analyses were considered with a 0.95 significance level.

## Results

### Cohort characteristics

A summary of the demographic and clinical characteristics for each group and detailed results of the group comparisons are presented in Table 1. Obstetric factors, multiple pregnancies, IUGR, and delivery by cesarean section were significantly more frequent in the PT group compared to FT (30.5 vs 1.5, 31 vs 2, and 68 vs 49%, respectively). As expected, PT infants differed significantly from FT group in weight, length, and head circumference at birth, as well as Apgar scores. Among the neonates admitted to NICU, only PT needed surfactant, ventilatory support and parenteral nutrition, with 7 (13%) infants needing mechanical ventilation >7 days and 4 (7.5%) parenteral nutrition >21 days.

**TABLE 1:** Detailed clinical and sociodemographic information for the 59 pairs of infants.

	Preterm group (*N* = 59)			Full-term controls (*N* = 59)			
		
	*NA*	*N* (%)	Median (IQR) [Range]	*NA*	*N* (%)	Median (IQR) [Range]	*p*
**Obstetric factors**							
Multiple pregnancy, *twins*		18 (30.5)			1 (1.7)		****
IUGR, *yes*	*4*	17 (30.9)¤		*2*	1 (1.8)¤		****
Maternal antenatal steroids, *given*		48 (81.4)			/		
Maternal antenatal magnesium sulphate, *yes*	*3*	25 (44.6)¤			/		
Mode of delivery, *cesarean section*		40 (67.8)			29 (49.2)		**
**Newborn characteristics at birth**							
Gestational age (*weeks)*			31.7 (28.8; 34.1) [23.7; 36.0]			40.1 (39.4; 41.1) [37.4; 42.3]	****
***GA grouping (by wGA)***							
Extremely PT (<*28 w)*		10 (16.9)			0		
Very PT (≥*28 to* < *32 w)*		23 (39.0)			0		
Moderate to late PT (≥*32 to* < *37 w)*		26 (44.1)			0		
Full-term birth (≥*37 w)*		0			59 (100)		
Sex, *male*		33 (55.9)			33 (55.9)		^#^
Birth weight (*kg*)			1.48 (1.10; 1.99) [0.54; 3.06]			3.47 (3.24; 3.74) [2.44; 4.14]	****
Birth length (*cm*)	*38*		44 (40; 47) [29; 53]	*11*		52 (50; 54) [46; 57]	****
Birth head circumference (*cm*)	*2*		29 (26; 31) [21.5; 35]	*1*		35 (34; 35.5) [31.5; 37]	****
**Neonatal period factors**							
Admitted in NICU, *yes*		51 (86.4)			2 (3.4)		****
Apgar score at first minute			7 (5; 9) [1; 10]			9 (8.5; 9) [1; 10]	****
Apgar score at fifth minute			9 (8; 9.5) [1; 10]			10 (10; 10) [6; 10]	***
Surfactant, *given*	*13*	22 (47.8)¤			/		
Mechanical ventilation > 7 days	*5*	7 (12.9)¤			0		***
Ventilatory support, *total days*	*5*		3.0 (1.0;19.5) [0.0;90.0]		/		
Oxygen needed, *total days*	*5*		1.0 (0.0;23.8) [0.0;99.0]		/		
Parenteral nutrition > 21 days	*5*	4 (7.4)¤			0		**
Parenteral nutrition, *total days*	*5*		5.0 (0.0;12.0) [0.0;29.0]		/		
Postnatal sepsis		14 (23.7)			4 (6.8)		*
Neonatal morbidities, *yes*	*15*	25 (56.8)¤			0		
CLD/O2 dependency at discharge	*5*	18 (33.3)/15 (27.7)¤			/		
Necrotizing enterocolitis	*5*	6 (11.2)¤			/		
Retinopathy of prematurity	*5*	2 (3.7)			/		
Any abnormal cUS	*16*	14 (32.5)¤			/		
**MRI scan around TEA**							
PMA (weeks) at MRI			41.3 (40.1; 42.1) [38.4; 44.9]			41.3 (40.2; 42.1) [38.3; 44.7]	^#^
Radiology score			2 (1; 3) [1; 3]			1 (1; 2) [1; 3]	*
1. Normal appearance for age		22 (37.3)			35 (59.3)		
2. Incidental findings with unlikely significance for clinical outcome or analysis		18 (30.5)			17 (28.8)		
3. Incidental findings with unlikely clinical significance but possible analysis significance		19 (32.2)			7 (11.9)		

Median IQR: interquartile range (25%; 75%); range: [minimum; maximum]. NA: data not available (number of subjects).

¤Percentage over the available data (see NA for missing data). Comparisons were performed with suitable tests (Wilcoxon rank sum test for ordinal and continuous variables; Fisher’s exact test for binary factors; Pearson’s Chi-squared test for non-binary nominal factors).

^#^No comparisons performed since these variables were used for pairing the full-term infants to preterm ones. cUS, cranial ultrasonography; CLD, chronic lung disease; FT, full-term birth; IUGR, intrauterine growth restriction; NICU, neonatal intensive care unit; PMA, post-menstrual age; PT, preterm birth; TEA, term equivalent age; wGA, weeks of gestational age.

*p*-value ≤ 0.0001 [****], ≤0.001 [***], ≤0.01 [**], < 0.05 [*], <0.1 [.], ≥0.1 [ns].

Morbidities linked to prematurity were seen in 25 PT infants (56.8% of the 44 with available data), including chronic lung disease for 18 infants (15 needing oxygen at discharge), 16 infants had an abnormality identified on cUS during NICU period, 6 had NEC and 2 had ROP. Of note, weight, length, and head circumference at TEA were not available in the dHCP dataset for all infants.

As would be expected, some differences were observed in Radiology scores at TEA between PT and FT babies, with more PT infants having a neuroradiology score equal to 3.

Selected clinical descriptors for the 4 infant subgroups included in the next descriptive analyses are presented in Supplementary Table 1.

### Neurodevelopmental outcome and characteristics at 18 months

Significant differences were observed in IMD scores reported at 18mCA (*n* = 96) (Supplementary Table 2), with PT families tending to live in more deprived areas than the FT ones (quintiles ≤ 3 for 64.3 vs 29.6%, respectively, *data not shown*).

**FIGURE 2:**
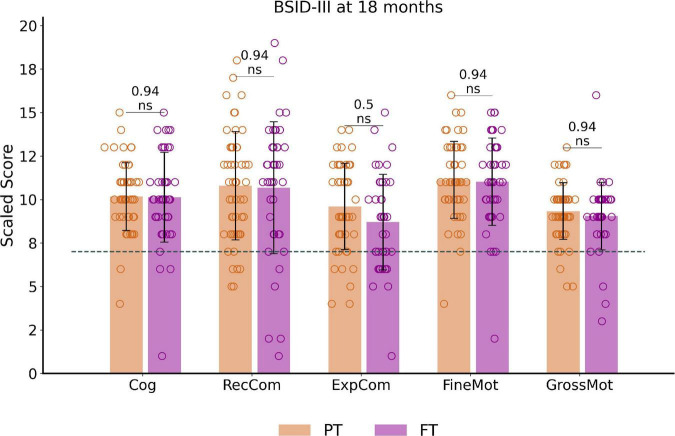
Outcome assessment at around 18 months of corrected age: BSID-III scaled scores distribution and comparisons between preterm and full-term infant groups. The dotted line corresponds to the pathological threshold, with scores <7 (<-1 SD) indicating a developmental delay. Of note, only one extreme PT (male, born at 27.6wGA) presented severe developmental delay (scores <-3SD) for cognitive and both communication scores (with fine motor score at –2SD and gross motor score on the norm values). Reported *p*-values come from *t*-tests corrected for multiple comparisons. See Supplementary Figure 1 for infant subgroup analysis using one-way ANOVA. BSID-III, Bayley Scales of Infant and Toddler Development, Third edition. PT, preterm; FT, full-term. Cog, cognitive; RecCom, receptive communication; ExpCom, expressive communication; FineMot, fine motor; GrossMot, gross motor scaled scores; ns, non-significant.

Among the 97 infants with available BSID-III data, no significant differences between PT and FT controls were observed for the corrected age at assessment. Paired *t*-tests on scaled scores showed no significant differences between PT and FT groups after correcting the results for multiple comparisons (Figure 2). BSID-III scaled scores across PT_EV_, PT_ML_ FT_EVCt_ and FT_MLCt_ subgroups (presented in Supplementary Figure 1) also showed no significant group effect.

Over the whole cohort, only a small number of infants showed scaled scores indicating a developmental delay (scores below 7 and corresponding to < -1SD), with no significant difference between PT and FT. These consisted of developmental delay in 30.9% of infants for expressive communication (*N* = 30, 19 PT), 14.4% for receptive communication (*N* = 14, 7 PT), 11.3% for gross motor (*N* = 11, 5 PT), 6.2% for fine motor (*N* = 6, 4 PT) and cognition (*N* = 6, 4 PT).

### Sensorimotor tract reconstructions

Visual inspection of the automated reconstructions for all tracts was performed on some randomly selected infants which allowed us to evaluate the quality of reconstructions for all the 15 tracts of interest, in a similar way across PT and FT infants. Examples of individual tract reconstructions and diffusion metric maps are shown in Figures 1C,D for a representative FT infant.

### Univariate tract-specific metrics

**FIGURE 3:**
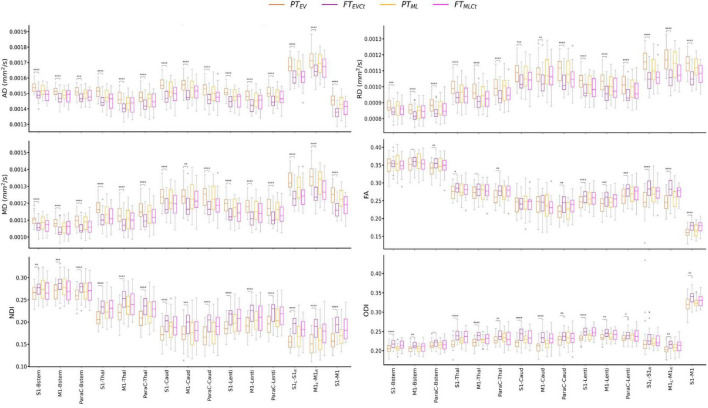
Diffusion metrics across tracts and cohort subgroups: extreme to very preterm group (PT_EV_, dark orange) compared to paired full-term controls (FT_EVCt_, dark purple), and moderate to late preterm group (PT_ML_, light orange) compared to paired controls (FT_MLCt_, light purple). Significances are results of the tract-specific paired *t*-tests between paired groups, corrected for multiple comparisons. Only the comparisons between PT_EV_ – FT_EVCt_ reached significance. Refer to Figure 1 legend for abbreviations and to Table 1 for *p*-value legend.

Tract-specific distributions of diffusion metrics across the 4 infant groups are presented in Figure 3. Visual assessment suggested important microstructural differences between groups. Results of the univariate analyses are presented in Supplementary materials: *Descriptive univariate analysis* section. Interestingly, we observed that AD, RD, MD (controlled for the effects of PMA at scan and WM microstructure) decreased with GA at birth in all tracts, while corrected FA, NDI and ODI increased.

The analysis of the relative percent difference in diffusion metrics between the PT and paired FT neonates allowed us to estimate a proxy of the maturational gap related to prematurity for each PT_EV_ and PT_ML_ group (Supplementary Figure 2). Visual inspection suggested a larger gap in the PT_EV_ subgroup than in PT_ML_, highlighting the effect of prematurity degree on tract microstructural characteristics. However, the observed variability between the metrics rendered the interpretation of different maturational patterns across tracts difficult, justifying the need for a multivariate approach.

### Mahalanobis distance of preterm subjects from the typical full-term profile

**FIGURE 4:**
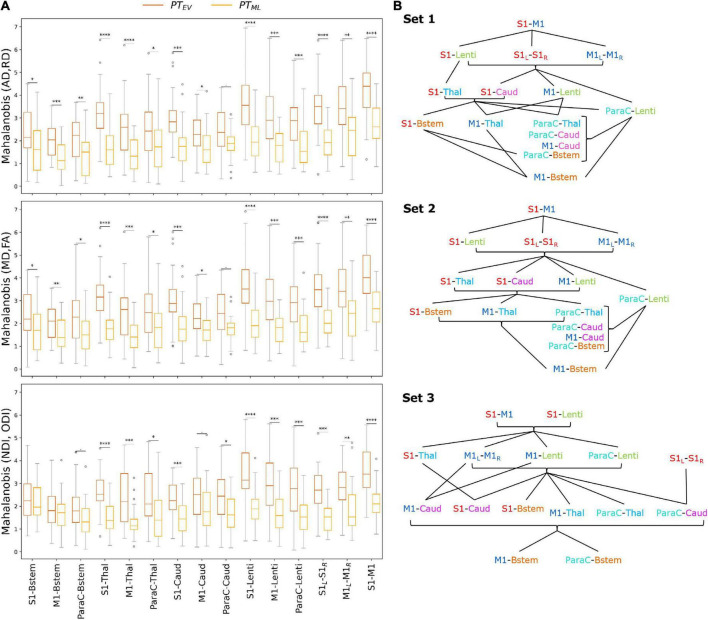
**(A)** Multi-metric Mahalanobis distance across tracts and PT subgroups (PT_EV_ and PT_ML_ with FT controls as reference) at TEA. The smaller the distance, the less the microstructural profile of the PT infant differs from the FT reference group. Note that the effect of prematurity is globally smaller for PT_ML_ than for PT_EV_ infants across the studied SM tracts. Significances are results of the comparison of Mahalanobis distances (PT_EV_ vs PT_ML_) with unpaired *t*-tests (*p*-values corrected for multiple corrections) for each set and each tract. For visualization purposes, outliers (mean ± 3SD) were removed (6 points for PT_EV_, 3 for PT_ML_ infants). **(B)** Order of the SM tracts in the PT_EV_ subgroup based on the Mahalanobis distance per metrics set (higher values on the top), highlighting the differential effect of prematurity on SM tracts microstructure. The lines represent the significant differences between tracts according to paired *t*-tests (corrected for multiple comparisons) over the PT_EV_ group (for visualization purposes, the statistical threshold was relaxed to *p* < 0.1). Metrics sets: 1 (AD, RD); 2 (MD, FA); 3 (NDI, ODI). Refer to Figure 1 legend for ROIs color code and abbreviations.

To explore the impact of prematurity on tract-specific microstructure, we computed multi-metric Mahalanobis distances of PT subgroups (PT_EV_ and PT_ML_ independently) to all FT infants as reference, using the 3 metric sets: *set 1* (AD and RD), *set 2* (MD and FA), and *set 3* (NDI and ODI) (Figure 4). For a given tract, computed Mahalanobis distance can be understood as a *maturational distance* for a given PT infant compared to the FT group.

For both PT subgroups (PT_EV_ and PT_ML_), all sets and all tracts, distances were highly significantly different from 0 as assessed by Wilcoxon tests corrected for multiple comparisons (all *p* < 0.001), suggesting that SM network microstructure is affected by prematurity, even moderate/late. Considering all PT infants, ANOVA modeling on distances for each set confirmed the expected effects of group, tract, and the interaction between group and tract for all three sets (Supplementary Table 3). As expected, the distance increased with the prematurity levels, with unpaired *t*-tests per tract comparing the two PT subgroups revealing higher distances in PT_EV_ than in PT_ML_ (Figure 4A). In addition, the tracts were not affected in the same manner: distances were different between PT_EV_ and PT_ML_ for all tracts except for ParaC-Caud in both DTI sets (1 and 2), and for set 3: S1-Bstem, M1-Bstem, ParaC-Bstem and M1-Caud.

#### Tract-specific effects of prematurity

To further evaluate the differential effect of prematurity on specific tracts, we subsequently compared distances for each pair of tracts through paired *t*-tests in each PT subgroup independently (Supplementary Figure 3). Many more significant tract-by-tract differences were observed in the PT_EV_ than in the PT_ML_ group (69 vs 26/105 for *set 1*; 70 vs 22/105 for *set 2*; 61 vs 23/105 for *set 3*), but the trends were rather consistent between the two PT subgroups.

Focusing on the *PT_EV_ subgroup*, the significant differences between tracts assessed by the paired *t*-tests allowed us to propose an ordering of the tracts based on the relative effects of prematurity on microstructural characteristics (Figure 4B). For *sets 1 and 2* (DTI sets), the orderings were highly similar, with, somewhat schematically, the following tracts showing the lowest to highest distances: (1) M1-Bstem; (2) ParaC-Bstem, S1-Bstem, M1-Thal, ParaC-Thal, M1-Caud, ParaC-Caud; (3) M1-Lenti, ParaC-Lenti, S1-Thal, S1-Caud; (4) S1-Lenti, S1_L_-S1_R_, M1_L_-M1_R_; (5) S1–M1. For *set 3* (NODDI set), the ordering showed schematically, from the lowest to highest distances of tracts: (1) M1-Bstem, ParaC-Bstem; (2) S1-Bstem, M1-Thal, ParaC-Thal, M1-Caud, ParaC-Caud, S1-Caud; (3) S1-Thal, M1-Lenti, ParaC-Lenti, S1_L_-S1_R_, M1_L_-M1_R_; (4) S1-Lenti, S1–M1 (Figure 4B).

Despite a few differences in the ordering of a couple of tracts, the results were quite consistent across the three sets and revealed a differential impact of prematurity on the SM tracts microstructure. Overall, the tract ordering based on maturational distances highlighted a coherent *caudo-rostral and central-to-periphery pattern*, with: the cortico*-Brainstem* tracts presenting the lowest distances and thus the least impact of prematurity; the cortico-*Thalamic* and cortico-*Caudate* tracts showing “intermediate” distances; the cortico-*Lenticular* tracts appearing with the highest distances among the cortico-subcortical tracts; and the *cortico-cortical* tracts revealing the highest prematurity impact. Inter-hemispheric tracts (S1_L_-S1_R_ and M1_L_-M1_R_) showed lower distances than the intra-hemispheric tracts (S1–M1). *S1 tracts* generally had higher distances than the M1 and ParaC tracts, both presenting similar profiles.

This approach of tract ordering based on the maturational distance related to prematurity was not considered for the *PT_ML_ subgroup* as tract pairwise comparisons were less systematically significant than in the PT_EV_ group and the ordering was more difficult to synthesize. For this subgroup, the distances of all tracts were more homogeneous (Supplementary Figure 3), which may be associated with a lesser effect of prematurity on the microstructural profiles of the SM tracts.

#### Tract-specific maturational distance associated with neurodevelopmental outcome

**FIGURE 5:**
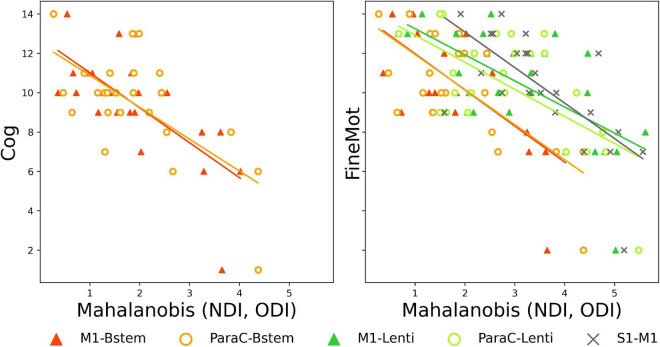
Significant correlations between Mahalanobis distances and neurodevelopmental scores at 18mCA, for NODDI set. Scatter plots of the significant Pearson correlations between tract-specific maturational distance related to prematurity at TEA in PT_EV_ group and BSID-III scores. Cog, cognitive; FineMot, fine motor scaled scores. Refer to Figure 1 legend for abbreviations.

Finally, we assessed whether maturational distances related to prematurity at TEA might be related to outcome (BSID-III scaled scores) at 18mCA, considering each PT subgroup independently. Pearson correlations showed significant results only in *PT*_EV_ group (*N* = 24), for *Set 3* (*NODDI*) (Supplementary Table 4) and for specific tracts: *M1-Bstem and ParaC-Bstem* distances were both negatively correlated with *Cognitive* scaled score and *Fine motor* score, while *M1-Lenti, ParaC-Lenti* and *S1*–*M1* tracts were also negatively correlated with *Fine motor* score (the lower the maturational distance, the higher the score and thus the better the outcome) (Figure 5). Interestingly, these five tracts showed different levels of distances over the PT_EV_ group, with M1- and ParaC-Bstem having a distance closest to 0, whereas S1–M1 was the tract with the highest distance; and M1- and ParaC-Lenti presented a similar and intermediate distance.

## Discussion

In this study, we considered an unprecedented set of primary SM cortico-cortical and cortico-subcortical tracts that are thought to underpin a wide range of early SM experiences. We observed significant differences in diffusion MRI derived metrics of WM microstructure between low-risk PT and FT infants at TEA. Multi-parametric assessment showed that the maturational gap differs with the prematurity level and across SM tracts, with alterations particularly affecting S1-related tracts and more rostral tracts. Importantly, these findings are of functional significance as correlations were also observed between specific measures of microstructural maturation within particular tracts and neurodevelopmental outcomes evaluated at 18mCA.

### Exploring the developing sensorimotor network microstructure with diffusion magnetic resonance imaging

#### A robust automated approach to delineate primary sensorimotor tracts in neonates

After meeting the challenge of extracting reliable individual SM tracts by optimizing the settings of an automated tractography-based approach benefitting from the dHCP multi-shell dMRI data, we explored the WM *maturational differences* of *low-risk* preterm infants at TEA compared to full-term neonates. We then quantified the microstructure of each tract by extracting DTI and NODDI-derived diffusion metrics using a weighting approach which privileges the core of the tract and avoids potential bias linked to some inter-individual differences in tract volumes.

#### Relevance of diffusion MRI models to characterize white matter maturation

DTI and NODDI models present different trade-offs between complexity, biological plausibility, robustness, and run-time duration (Jelescu and Budde, 2017). Despite its widespread use in most studies of WM development (Dubois et al., 2014; Ouyang et al., 2019), DTI-derived metrics can be affected by several microstructural features and lack specificity to disentangle the complex properties of voxels containing crossing, kissing and fanning fibers (Zhang et al., 2012; Jeurissen et al., 2013). In contrast, NODDI allows a more sophisticated and biologically plausible multi-compartment model, relevant for developmental studies (Chang et al., 2015; Genc et al., 2017; Mah et al., 2017; Kimpton et al., 2021), but requires multi-shell data and increased processing time. Although potentially sub-optimal, we opted for default settings of diffusivities in the NODDI model, which were optimized for the adult WM but not for infants (Guerrero et al., 2019). In the absence of gold standards for infant-specific NODDI fitting to evaluate the metric maps, this was performed to maintain some consistency with previous studies (Guerrero et al., 2019; Fenchel et al., 2020).

Overall, the resulting metrics maps were consistent among PT and FT subjects, and metric differences across tracts were largely coherent between DTI and NODDI results in all infants. Once controlled for the effects of PMA at scan and global WM microstructure, we observed that AD, RD, MD decreased with GA at birth in all tracts, while FA, NDI and ODI increased. Globally, MD differences across tracts presented an opposite pattern to NDI (Kimpton et al., 2021), and differences in RD were highly similar to MD in all tracts, and opposite to FA in cortico-subcortical tracts, confirming that variations in MD and FA are likely largely driven by RD. Such diverse profiles across tracts might result from differences in both intrinsic microstructure (similar to adults) and in maturation (according to different myelination stages across tracts) (Dubois et al., 2014). During WM development, MD tends to decrease and NDI to increase with the growth of fibers and membranes, acting as barriers to the random water motion, while FA tends to increase, reflecting several factors including the presence of compact fiber tracts and increasing myelination (Beaulieu and Allen, 1994; Batalle et al., 2017; Kimpton et al., 2021). ODI describes the orientational dispersion of fibers within a tract, which is highly variable across tracts and likely changes during the growth and maturation of crossing fibers (Raghavan et al., 2021). Interestingly, in a few tracts, we did not observe significant relationship between FA or ODI (corrected for PMA at scan and WM residuals) and GA at birth, suggesting that both metrics might be less sensitive to detect subtle variations of microstructure in the settings of this study. Further analyses (beyond the scope of this study) could be performed to evaluate the potential correlations between all these metrics, as performed in previous studies (Kunz et al., 2014).

Moreover, the results of the maturational distance analyses underlined a high coherence between DTI sets which seemed fairly intuitive and in line with previous studies (Li et al., 2022). The differences observed between the DTI and NODDI sets (with NODDI set presenting more compact and roughly lower Mahalanobis distances values across tracts, with subtle variations in the main order of distances) suggested that NODDI metrics provide complementary information, probably due to their differing sensitivity to neurites growth and maturation. Thus, in line with previous studies (Batalle et al., 2017; Kimpton et al., 2021), our results highlighted the complementarity of these models and confirmed the relevance of NODDI-derived metrics for the study of WM microstructure maturation in the context of prematurity.

### Studying the effects of prematurity on sensorimotor network maturation at term-equivalent age

#### Hypotheses about the differential effect of prematurity across primary sensorimotor tracts

In contrast to the majority of previous studies, we focused on low-risk PT infants without overt brain abnormality at TEA, which is representative of the majority of children now born preterm in developed countries. Despite the presence of specific clinical risk factors in some of the PT infants (e.g., morbidities related to prematurity), the absence of a significant difference in the BSID-III outcome at 18 months of age between PT and FT infants corroborated that the included PT infants were at low-risk for neurodevelopment impairment.

In this study, we hypothesized that WM microstructure within SM tracts would show a significant maturational delay at TEA in PT infants compared to FT neonates, with distinct patterns as a function of GA at birth and across cortico-subcortical and cortico-cortical tracts. This was based on the assumption that early peripheral stimuli are essential for the emerging SM network maturation, and that preterm birth is associated with modified SM stimuli and experiences, notably related to numerous and various procedures in NICU (Mörelius et al., 2006; Gibbins et al., 2008), which might have a differential effect on somatosensory and motor systems (Duerden et al., 2018; Schneider et al., 2018; Jones et al., 2022). Of direct relevance, previous studies have reported either higher tactile sensitivity in PT infants at TEA (André et al., 2020), tactile hyporeactivity, lower brain responses (Maitre et al., 2017) and/or undifferentiated integration of nociceptive versus non-nociceptive stimuli (Fabrizi et al., 2011), in association with WM abnormalities (Brummelte et al., 2012; Zwicker et al., 2013).

#### Approaching the effects of prematurity on WM microstructure with univariate analyses

Univariate dMRI approaches, based on individual derived metrics, are commonly applied to reveal WM developmental changes in the neonatal brain (Kunz et al., 2014; Ouyang et al., 2019). These analyses were thus used to evaluate the effects of several factors on the diffusion metrics measured in SM tracts.

Firstly, these were not related to the infants’ sex in our cohort. Combined with previous studies which showed inconsistent results (Pannek et al., 2014; Barnett et al., 2018; Kimpton et al., 2021), this observation suggests that sex effects may vary according to the studied brain regions. Surprisingly, we also observed no effect of main perinatal clinical risk factors (including preterm morbidities), despite numerous studies describing associations of WM abnormalities with obstetric, neonatal and postnatal factors (Pogribna et al., 2013; Brouwer et al., 2017; Barnett et al., 2018; Parikh et al., 2021), and with exposure to cumulative risk factors (Barnett et al., 2018). These negative results might be partly due to the specific study of low-risk PT infants.

In line with previous literature, we observed that diffusion metrics –even measured at TEA– were dependent on PMA at MRI (de Bruïne et al., 2011; van Pul et al., 2012; Kimpton et al., 2021) and were influenced by prematurity (Kunz et al., 2014; Kelly et al., 2016a; Batalle et al., 2017; Thompson et al., 2019; Dibble et al., 2021; Kimpton et al., 2021), with PT_EV_ infants showing more “immature” microstructural profiles (higher AD, RD, MD, lower FA, NDI, ODI) than PT_ML_ and FT infants. Interestingly, when group analyses were performed at the tract level, PT_ML_ showed no difference with the FT paired group, suggesting that the specific SM tracts studied here might not contribute significantly to the well-described whole-brain WM diffusion abnormalities in moderate-late PT (Kelly et al., 2016a; Thompson et al., 2019).

#### Evaluating the maturational distances related to prematurity with a multivariate approach

We then aimed to evaluate the maturational gap between PT infants and their full-term peers, based on SM tracts microstructural characteristics at TEA. Although univariate dMRI approaches allow some inference about the effects of prematurity on SM tract microstructure, they cannot reflect the inter-related complexity of processes involved in early brain maturation (Kostović et al., 2019), and are limited by the difficulties interpreting findings related to single metrics which are sensitive to different underlying microstructural properties and maturational processes. Also, quantifying the maturational degree across regions requires comparison of an infant’s data with a mature reference to account for “intrinsic” microstructural differences (Dubois et al., 2014). To overcome these limitations, we implemented an original multivariate approach already validated in neonatal and pediatric data (Kulikova et al., 2015; Dean et al., 2017; Li et al., 2022), that took advantage of the complementary information described by different DTI and NODDI metrics, to enable better characterization of SM tract maturation and the effects of prematurity as compared to typical development. Multivariate *Mahalanobis distance* was calculated in respect to a reference group (FT neonates) which provided typical values for the given tract. Importantly, this approach also allowed to take into consideration both the inherent variability of the diffusion metrics across tracts in the FT group and the correlations between these metrics. For each tract, the resulting maturational distance related to prematurity could be interpreted as a developmental gap between a PT infant at TEA and the FT control group.

#### Highlighting the tract-specific effects of prematurity on SM network

Focusing on the PT_EV_ group, the comparison of distances across tracts highlighted the *differential impact of prematurity* on the SM tracts at TEA. For all sets of diffusion metrics, the impact increased in a *caudo-rostral and central-to-peripheral manner* following the typical progression of WM growth and myelination during infancy (Yakovlev and Lecours, 1967; Dubois et al., 2014) and within the CST tract (Kimpton et al., 2021), suggesting that early maturing tracts are less impacted. Furthermore, while this spatial pattern is globally consistent with previous studies of preterm infants (Wu et al., 2017; Knight et al., 2018), our results raise several interesting points regarding the functional role of the different SM tracts and related GM structures during development.

Firstly, we observed a differential impact of prematurity among tracts related to different cortical seeds. *S1-subcortical* tracts were systematically more impacted than M1- and ParaC-subcortical tracts. This suggests that S1 tracts may have a *specific vulnerability* to the deleterious effects of prematurity, possibly due to the altered SM perceptions and experiences as a result of early exposure to the ex-utero environment. Alternatively, the observed differences may reflect compensatory faster and/or more efficient maturational “catch-up” mechanisms in the M1/ParaC-subcortical tracts during the first post-natal weeks after preterm birth.

The similar profiles seen in the *ParaC-subcortical* tracts and related *M1-subcortical* tracts are less straightforward to interpret as the paracentral lobule includes both motor and somatosensory regions. Given the somatotopic organization of S1 and M1, this could suggest that connections related to the lower limb representations are less impacted by prematurity, which may be linked to the possible advanced maturation of these representations at early ages (Devisscher et al., 2021). A further possible explanation is that a greater number of motor fibers than sensory fibers were included in ParaC- tracts.

Secondly, the prematurity impact was also variable across sub-cortical related tracts. Of these, the *cortico-Brainstem* tracts appeared the least impacted. As these mainly correspond of CST fibers that myelinate early, notably at the level of the PLIC (Dubois et al., 2014; Kulikova et al., 2015; Kimpton et al., 2021), this is consistent with connections that have more advanced maturation at the time of birth being less vulnerable to prematurity (Wu et al., 2017). In agreement with the acknowledged vulnerability of thalamocortical connections following preterm birth (Ball et al., 2013a), the *cortico-Thalamic* tracts showed higher impact than cortico-Brainstem tracts, giving an “intermediate” profile compared to other studied tracts. The specific functional role of the thalamus, with essential input and output projections to the different SM regions, might help to modulate this vulnerability compared to other sub-cortical structures (Duerden et al., 2018; Schneider et al., 2018). Similarly, the *cortico-Caudate* tracts showed “intermediate” profile. This might result from an interplay between the high vulnerability of the caudate nuclei to prematurity (Nosarti et al., 2014; Back, 2015; Loh et al., 2017) and the adverse effects on the major efferent projections (McClendon et al., 2014) compared with more “preserved” afferent connections (from SM cortices). The *cortico-Lenticular* tracts systematically presented the maturational distance profile with the greatest impact of prematurity, suggesting their specific vulnerability. In addition to the known structural consequences of preterm birth on BG growth (Loh et al., 2017, 2020), different hypotheses can be proposed to explain this specific profile, especially knowing the anatomo-functional particularities of these tracts. As the dissected tracts include both (afferent) *cortico-putaminal* and (efferent) *pallido-cortical* fibers, the observed alteration may involve both the input (putamen) and output (GPi) structures of the BG, which have different functions in cortico-BG loops. We hypothesize that maturation of the *efferent* pallido-cortical fibers is specifically altered by prematurity, with functional effects on information reaching SM cortices, which might secondarily induce alterations of the descending cortico-striatal and cortico-pallidal fibers.

Finally, we observed the highest impact of prematurity in the *cortico-cortical* tracts, suggesting a particular vulnerability of these rostral structures, in line with the well-described caudo-rostral maturational pattern. *Inter*-hemispheric tracts presented lower impact than *intra*-hemispheric S1–M1 tracts, in line with the late and protracted maturation of such short-range connections (Kostović et al., 2019).

While interesting, the results should be interpreted cautiously given the limitations of diffusion MRI and tractography with relation to the image spatial resolution and the size of neonatal structures, and the presence of crossing fibers notably at the level of the corona radiata. However, the rare high-quality of dHCP neonatal data, the use of HARDI acquisition, the consistency of the tract’s delineation and the multivariate approach allowed us to overcome, at least partially, these limitations. In the future, it would be interesting to further investigate whether the vulnerability of SM tracts to prematurity is stable over development or whether “catch-up” development is present for some tracts, either before or after TEA. This would require the longitudinal evaluation of maturational distances defined with similar settings.

### Relating the early microstructure of sensorimotor tracts with neurodevelopmental outcome

The final aim of this study was to investigate the relationship between SM microstructural characteristics at TEA and neurodevelopmental outcome at 18mCA. In the specific low-risk preterm cohort studied, no substantial developmental delay or specific disability was expected, as confirmed by the results in terms of BSID-III scores.

Nevertheless we hypothesized that correlations would exist between diffusion metrics profiles and BSID-III scores based on previous studies showing that, even in the absence of overt brain lesions, neonatal microstructural WM measures are associated with neurodevelopmental outcome in toddlers and children (van Kooij et al., 2012; Duerden et al., 2015; Barnett et al., 2018; Girault et al., 2019; Kelly et al., 2020; Pannek et al., 2020; Parikh et al., 2021). In particular, reduced neonatal FA (especially in the PLIC) has been associated with delayed psychomotor development and motor disability at different ages (Rose et al., 2007; Skranes et al., 2007; De Bruïne et al., 2013; Groeschel et al., 2014; Kelly et al., 2016b), and neonatal NODDI metrics have been found to relate to later neurodevelopmental outcomes (Kelly et al., 2016b; Young et al., 2019).

Our results showed negative correlations in PT_EV_ infants between maturational distances and *Cognitive* and *Fine motor scaled scores* for a number of tracts in the *NODDI set* only. This suggested that the early microstructural information as modeled by NODDI is more sensitive than DTI based metrics such as FA for detecting subtle WM tract alterations related to later neurodevelopmental impairments in preterm infants (Batalle et al., 2017; Kimpton et al., 2021). Moreover, early SM tract microstructure was further correlated with *cognitive* outcomes, confirming essential developmental interactions between the SM system and higher-order functions, and the common clinical overlap of motor and cognitive impairments in the PT population.

Regarding the tracts concerned, we first observed that *fine motor* score was related to five tracts with different maturational distance profiles: *M1-Brainstem and ParaC-Brainstem* which were the least impacted by prematurity; *M1-Lenti and ParaC-Lenti* with intermediate profile; and *S1–M1* with the greatest impact. This suggested that the degree of maturational gap at TEA by itself is not the only factor explaining motor outcomes.

In the light of our results showing the high vulnerability of *lenticular* tracts to prematurity, it is not surprising that microstructural alterations in the motor tracts connected to this key BG structure may underpin early SM impairments with further consequences on fine motor skill acquisition (Leisman et al., 2014). Likewise, as intra-hemispheric SM connections contribute to improve SM integration and functions, the correlation observed for *S1–M1* tract suggests that early impact of prematurity on these tracts may alter later neuromotor development.

Maturational distances for *M1-Brainstem and ParaC-Brainstem* tracts were also correlated with *cognitive* scores, suggesting that early microstructural alterations in these tracts might have global functional consequences, beyond motor skills. Nevertheless, further studies are needed to better understand the involvement in these developmental domains of the brainstem, a complex structure that plays an essential role as a relay for a large number of connections from the whole nervous system, in addition to the functions associated with its many GM sub-structures.

The observed relationships in PT_EV_ infants between SM tract microstructure at TEA and outcome at 18mCA are of particular interest in the context of prematurity, as even low-risk populations are at increased risk of –sometimes subtle– neuromotor impairments (e.g., developmental coordination disorder) (Edwards et al., 2011; Spittle and Orton, 2014; Zwicker, 2014; Groeschel et al., 2019). These disorders are generally not visible enough to be diagnosed until much later (often at school age) (Williams et al., 2010; de Jong et al., 2012; Van Hus et al., 2014), which underlines the need for early diagnostic biomarkers. Thus, the specific impact of prematurity on the five primary SM tracts previously mentioned should be further explored, in order to investigate their potential value as early markers of motor and/or other neurodevelopmental disorders such as developmental coordination disorder.

Nevertheless, whilst relating early brain markers and long-term outcome has important clinical relevance, previous studies have described that environmental factors (e.g., socio-familial) could explain the greatest part of interindividual variability in neurodevelopment later in childhood, with the influence of perinatal risk factors diminishing over time (Thompson et al., 1998; Miceli et al., 2000; Anderson and Doyle, 2008; Linsell et al., 2015). Thus, future studies should incorporate more accurate predictive models to intend to approach the complex relationship between early brain characteristics, environmental factors and outcome.

## Conclusion

Using an unprecedented combination of diffusion MRI data and innovative analysis methods, our results confirmed that prematurity impacts early microstructural development of the primary SM network, even in low-risk preterm infants. We further found that these effects differ according to the level of prematurity and across the SM tracts, with the most rostral tracts as well as those involving S1 showing the greatest vulnerability to prematurity at TEA. Our study also showed the complementarity between DTI and NODDI models as well as the interest of using multiparametric approaches for assessing maturational processes and microstructural developmental differences. Longitudinal studies incorporating earlier MRI evaluation as well as behavioral follow-up through to later childhood would provide a better understanding of the impact of early-life disturbances in SM tracts microstructure on neurodevelopmental outcomes.

## Data availability statement

Publicly available datasets were analyzed in this study. This data can be found here: http://www.developingconnectome.org.

## Ethics statement

The studies involving human participants were reviewed and approved by UK NHS Research Ethics Committee (14/LO/1169, IRAS 138070). Written informed consent to participate in this study was provided by the participants’ legal guardian/next of kin.

## Author contributions

SN, AG, LH-P, TA, and JD: conceptualization. SN, AG, YL, and JD: methodology. SN, AG, TA, and JD: resources and investigation. SN and AG: data curation. SN, AG, and JD: validation and formal analysis, visualization, and writing – original draft. AG and YL: software. TA and JD: supervision. SN, AG, YL, LH-P, TA, and JD: writing – review and editing. All authors contributed to the article and approved the submitted version.

## Footnotes

1
http://www.developingconnectome.org/

2
https://biomedia.github.io/dHCP-release-notes/download.html

## Abbreviations

AD: axial diffusivity
BG: basal ganglia
BSID-III: Bayley Scales of Infant and Toddler Development 3rd edition
Bstem: brainstem
Caud: caudate nucleus
CLD: chronic lung disease
CSF: cerebrospinal fluid
CST: corticospinal tract
cUS: cranial ultrasonography
dHCP: developing Human Connectome Project
dMRI: diffusion-weighted MRI
DTI: diffusion tensor imaging
FA: fractional anisotropy
FDR: false discovery rate
FT: full-term born infants [FT_EVCt,_ “extreme to very preterms” matched FT controls FT_MLCt,_ “moderate to late preterms” matched FT controls]
GA: gestational age
GM: gray matter
IMD: index of multiple deprivation
IUGR: intrauterine growth restriction
Lenti: lenticular nucleus
M1: lateral portion of the primary motor cortex (precentral region)
mCA: months of corrected age
MD: Mean diffusivity
MRI: magnetic resonance imaging
NDI: neurite density index
NEC: necrotizing enterocolitis
NICU: neonatal intensive care unit
NODDI: neurite orientation dispersion and density imaging
ODI: orientation dispersion index
ParaC: medial portions of the primary sensori-motor cortices (paracentral region)
PMA: post-menstrual age
PT: preterm infants [PT_EV_, extreme to very preterms PT_ML_, moderate to late preterms]
RD: radial diffusivity
ROIs: regions of interest
ROP: retinopathy of prematurity
S1: lateral portion of the primary somatosensory cortex (postcentral region)
SES: socio-economic status
Thal: thalamus
TEA: term-equivalent age
wGA: weeks of GA
WM: white matter.
