# Supplementary material for: Early structural connectivity within the sensorimotor network: Deviations related to prematurity and association to neurodevelopmental outcome
Source: Front Neurosci. 2022 Nov 25;16:932386. doi: 10.3389/fnins.2022.932386 (PMC9732267; doi:10.3389/fnins.2022.932386)
Supplement: Supplementary file 1 [file Data_Sheet_1.docx]

# Supplementary Materials

**Table SupT1. Clinical factors for the 4 infants’ sub-groups used for the adjustments in descriptive models.** Preterm infants (PT) were subdivided into those born extremely to very preterm (PT_EV_) or moderate to late preterm (PT_ML_) and their corresponding full-term (FT) controls. ​

|  | **PT_EV_**  (N=33) | **FT_EVCt_**  (N=33) | **PT_ML_**  (N=26) | **FT_MLCt_**  (N=26) |
| --- | --- | --- | --- | --- |
| GA at birth (*weeks*)  *Median (IQR) [range]* | 29.0  (27.4; 30.7)  [23.7; 31.9] | 40.4  (39.9; 41.1)  [37.4; 42.3] | 34.3  (33.2; 35.1)  [32.3; 36.0] | 40.1 (39.4;40.8) [37.4;42.1] |
| Sex, *male* | 18 (55%) | 18 (55%) | 15 (58%) | 15 (58%) |
| Multiple Pregnancy, *twins* | 9 (27%) | 0 | 9 (35%) | 1 (4%) |
| IUGR | 10 (33%)  *NA: 3* | 0  *NA: 1* | 7 (28%)  *NA: 1* | 1 (4%)  *NA: 1* |
| Preterm Morbidities | 21 (64%) | 0 | 4 (36%)  *NA: 15* | 0 |
| Parenteral Nutrition >21days | 4 (12%) | 0 | 0  *NA: 5* | 0 |
| PMA at MRI (*weeks*)  *Median (IQR) [range]* | 41.3  (40.1; 42.3)  [38.4; 44.9] | 41.3 (40.3;42.3) [38.3;44.7] | 41.1 (40.1;42.0) [38.9;44.1] | 41.1 (40.1;42.0) [38.9;44.1] |

Refer to *Table 1* legend for abbreviations.

**Table SupT2. Cohort characteristics and outcome assessment at around 18 months of corrected age.**

|  |  | **Preterm infants**  (*n*=59) | | **Full-term controls**  (*n*=59) | |  |
| --- | --- | --- | --- | --- | --- | --- |
|  |  | *NA* |  | *NA* |  | *p* |
| **Sex**, male | N (%) | *15* | 23 (52.3) ¤ | *6* | 30 (56.6) ¤ | *#* |
| **Age at assessment** (*months*) | Median  (IQR)  [Range] | *15* |  | *6* |  |  |
| *Chronological* age |  |  | 20.4  (19.8;21.1) [18.9;23.4] |  | 18.2  (17.8;18.7) [17.3;19.8] | *** |
| *Corrected* age |  |  | 18.4  (18.1;18.7) [17.7;20.8] |  | 18.2  (17.9;18.6) [17.3;19.8] | ns |
| **Family socioeconomic status:**  Index of multiple deprivation (IMD) | Median  (IQR)  [Range] | *17* | 17.8  (11.1;29.7) [2.7;48.3] | *5* | 26.6  (18.2;33.8) [4.2;46.4] | * |
| BSID-III: ***Scaled* scores** | Mean (SD)  [range] | *15* |  | *6* |  |  |
| Cognitive |  |  | 10.1 (2.6) [1.0;15.0] |  | 10.2 (2.0) [4.0;15.0] | ns |
| Receptive communication |  |  | 10.7 (3.8) [1.0;19.0] |  | 10.8 (3.1) [5.0;18.0] | ns |
| Expressive communication |  |  | 8.7 (2.8) [1.0;15.0] |  | 9.6 (2.5) [4.0;14.0] | ns |
| Fine motor |  |  | 11.0 (2.5) [2.0;15.0] |  | 11.1 (2.2) [4.0;16.0] | ns |
| Gross motor |  |  | 9.0 (2.0) [3.0;16.0] |  | 9.3 (1.6) [5.0;13.0] | ns |

¤ percentage over the available data (see NA for missing data). Comparisons for ages at assessment and IMD scores were performed with Wilcoxon rank sum test. #: no comparisons done for sex (used for pairing the full-term controls). *p*-values for BSID-III scores were obtained from t-tests corrected for multiple comparisons. Refer to *Table 1* legend for abbreviations. See *Figure 2* for BSID-III scores distributions and graphical comparison between groups.


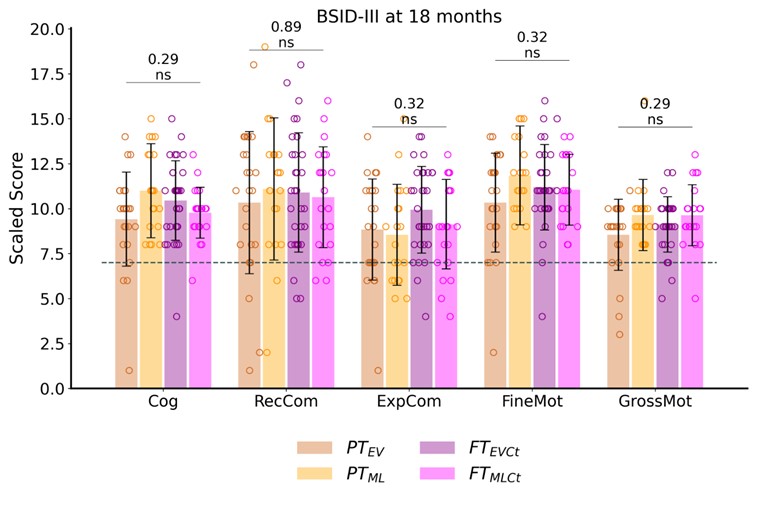
**Figure SupF1**. **Outcome assessment at around 18 months of corrected age: BSID-III scaled scores across subgroups** (PT_EV_, PT_ML_, FT_EVCt_, and FT_MLCt_), with the results of one-way ANOVA and *p*-values indicating no significant group effect. The dotted line indicates the scores threshold indicating a developmental delay (scores < -1 SD): scaled scores < 7. Cog: cognitive; RecCom: receptive communication, ExpCom: expressive communication; FineMot: fine motor, GrossMot: gross motor scores. ns: not significant.


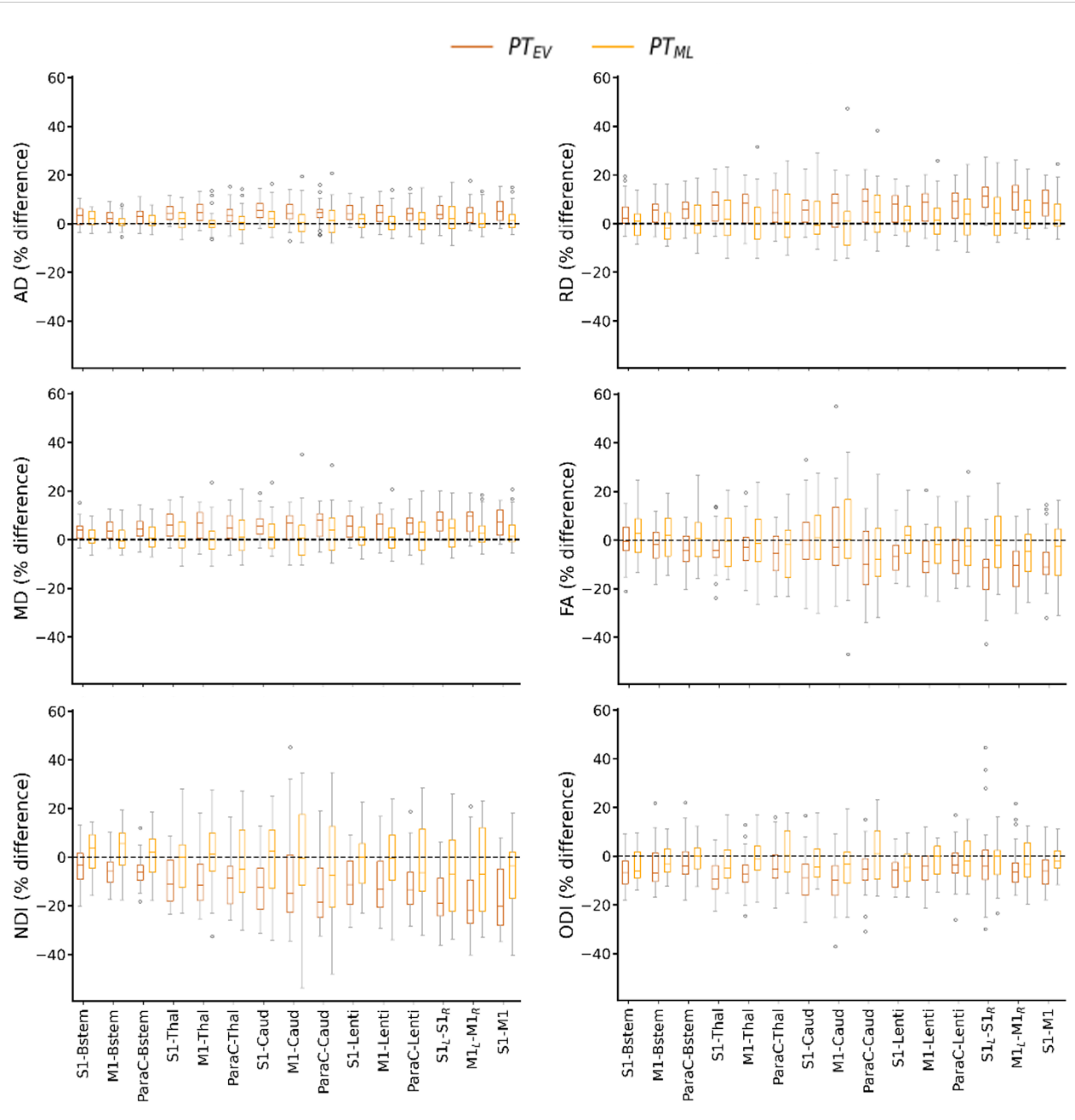


**Figure SupF2.** Relative percent difference in diffusion metrics between PT_EV_ vs FT_EVCt_ and PT_ML_ vs FT_MLCt_ groups (each PT infant being compared to his/her paired FT newborn) for each SM tract. Refer to *Figure 1* legend for abbreviations.

**Table SupT3.** ANOVA model studying effects of tract and PT subgroup on Mahalanobis distances, for each set. Refer to *Table 1* for *p*-value legend.

|  | **Set 1 (AD, RD)** | **Set 2 (MD, FA)** | **Set 3 (NDI, ODI)** |
| --- | --- | --- | --- |
| Tract | **** | **** | **** |
| Group | **** | **** | **** |
| Tract : Group | **** | **** | **** |

**
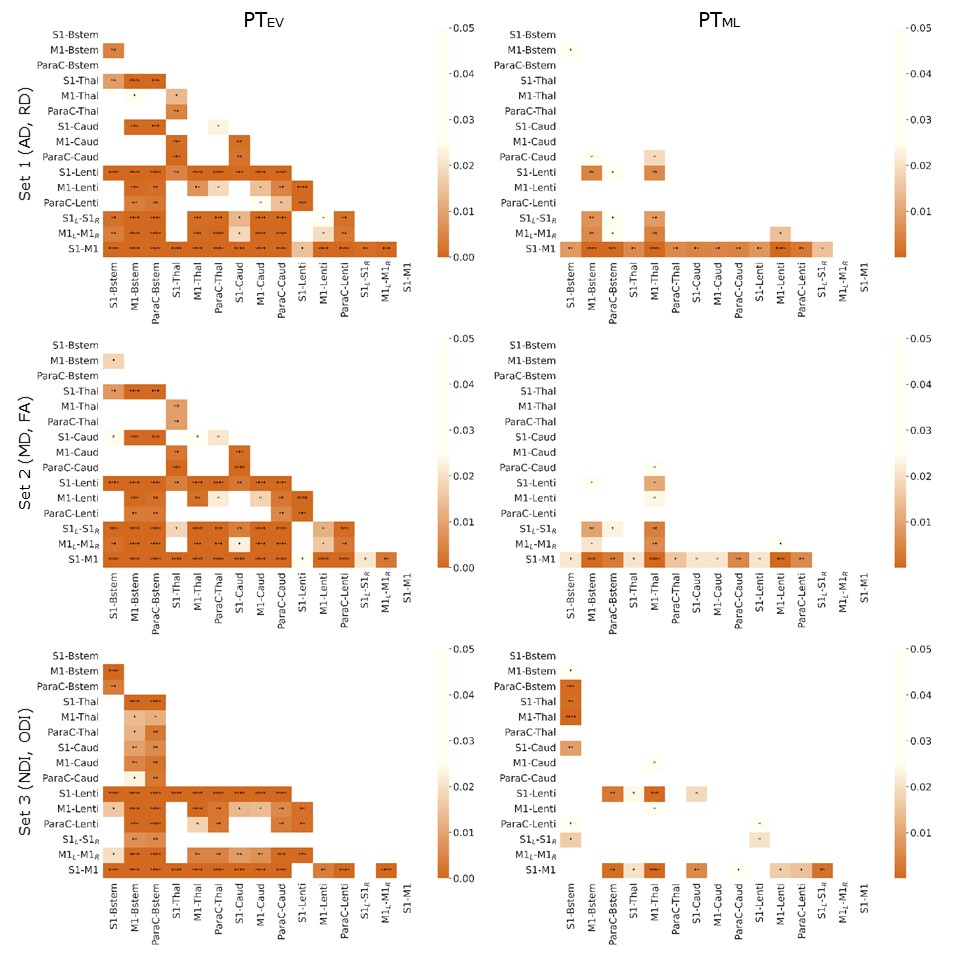
**

**Figure SupF3. Differential effect of prematurity on specific SM tracts.**Heatmap of p-values from paired t-tests (corrected for multiple comparisons) of Mahalanobis distances for each pair of SM tracts, for each set of metrics, in each PT subgroup independently (PT_EV_ on the left, PT_ML_ on the right). Legend depicts *p*-values with respect to heat intensity, with darker colours representing greater statistical significance and white no significant differences. Refer to *Figure 1* legend for abbreviations and to *Table 1* for p-value legend.

**Table SupT4. Pearson correlation analyses between Mahalanobis distance and BSID-III scaled scores.** Only PT_EV_ results for *set 3* (NODDI) are shown (p-values corrected for multiple comparisons), as no significant correlations were found in *sets 1* and *2.*

|  | **PT_EV_ - Set 3 (NDI, ODI)** | | | | | | | | | |
| --- | --- | --- | --- | --- | --- | --- | --- | --- | --- | --- |
|  | **Cognitive** | | **Receptive Com.** | | **Expressive Com.** | | **Fine Motor** | | **Gross Motor** | |
| Tract | r | *p* | r | *p* | r | *p* | r | *p* | r | *p* |
| S1-Bstem | -0.51 | ns | -0.46 | ns | -0.31 | ns | -0.56 | . | 0.12 | ns |
| **M1-Bstem** | -0.7 | * | -0.55 | . | -0.5 | ns | -0.68 | * | 0.08 | ns |
| **ParaC-Bstem** | -0.66 | * | -0.45 | ns | -0.47 | ns | -0.69 | * | 0.03 | ns |
| S1-Thal | -0.35 | ns | -0.22 | ns | -0.13 | ns | -0.48 | ns | 0.33 | ns |
| M1-Thal | -0.5 | ns | -0.43 | ns | -0.31 | ns | -0.58 | . | 0.08 | ns |
| ParaC-Thal | -0.34 | ns | -0.32 | ns | -0.25 | ns | -0.54 | ns | 0.1 | ns |
| S1-Caud | -0.41 | ns | -0.12 | ns | 0.02 | ns | -0.31 | ns | 0.13 | ns |
| M1-Caud | -0.27 | ns | -0.15 | ns | -0.12 | ns | -0.33 | ns | 0.12 | ns |
| ParaC-Caud | -0.35 | ns | -0.39 | ns | -0.33 | ns | -0.42 | ns | -0.02 | ns |
| S1-Lenti | -0.52 | ns | -0.48 | ns | -0.36 | ns | -0.57 | . | 0.15 | ns |
| **M1-Lenti** | -0.46 | ns | -0.51 | ns | -0.4 | ns | -0.62 | * | 0.02 | ns |
| **ParaC-Lenti** | -0.45 | ns | -0.47 | ns | -0.41 | ns | -0.62 | * | -0.02 | ns |
| S1_L_-S1_R_ | -0.01 | ns | -0.02 | ns | 0.11 | ns | -0.06 | ns | 0.17 | ns |
| M1_L_-M1_R_ | -0.47 | ns | -0.49 | ns | -0.43 | ns | -0.56 | . | -0.03 | ns |
| **S1-M1** | -0.56 | . | -0.54 | ns | -0.35 | ns | -0.68 | * | 0.1 | ns |

Refer to *Table 1* for p-value legend and to *Figure 1* for tracts abbreviations.

## *Descriptive univariate analysis*

*For the sake of clarity, the tables and figures from this section are referred to as Uni in the text.*

To prepare the settings of multivariate analyses, we performed three preparatory univariate analyses on diffusion metrics.

1) We first aimed to evaluate whether differences between left and right tracts were important to consider, and to check that differences were present between groups. As we aimed to perform a single analysis for all tracts together, we add to further introduce this factor.

Considering all tracts except interhemispheric connections, we assessed whether different factors (namely the tract, the hemisphere, and the infant group) have an effect on each diffusion metric by implementing a global ANOVA model. The results **(Table UniT1. A)** confirmed the expected significant effects of tract microstructure and group, as well as the interaction between both, except for FA where the group did not reach the significance level. A significant effect of hemisphere was further observed for FA, RD, and ODI metrics as well as interaction between tract and hemisphere, uncovering asymmetries between left and right tracts. However, given that no significant interaction was observed between hemisphere and group for any of the six evaluated metrics, we decided not to further explore these differences between left and right tracts, and to consider *averaged diffusion metrics for left and right tracts* in all subsequent analyses**.**

Additionally, the differences between groups of interest (PT_EV_ vs FT_EVCt_; PT_ML_ vs FT_MLCt_) was studied post-hoc with paired t-tests (over all tracts) corrected for multiple comparisons across the metrics and per each tract independently**.** The groups analyses which considered all tracts corroborated the existence of significant differences between PT infants and their matched FT controls, with higher AD, RD and MD metrics, opposed to lower FA, NDI, and ODI metrics in PT groups compared to FT, especially in the PT_EV_ vs FT_EVCt_ (**Table UniT1. B**). This suggested that the more preterm the infant is, the more “immature” microstructural characteristics are. Group comparisons for PT_EV_ vs FT_EVCt_ per tract are presented in **Table UniT2** (group comparisons between PT_ML_ vs FT_MLCt_ were not significant).

**Tables UniT1. A.** ANOVA model studying effects of tract, hemisphere, infant group (considering *extreme to very preterms* (PT_EV_) / *moderate to late preterms* (PT_ML_) and their paired controls (FT_EVCt_ and FT_MLCt_, respectively), and their interactions on each diffusion metric. **B.** Paired t-test comparisons between PT_EV_, PT_ML_, and paired FT controls for each diffusion metric, over all tracts (p-values corrected for multiple comparisons).

Refer to *Table 1* for abbreviations and *p*-values significance.

**A.**

|  | **AD** | **RD** | **MD** | **FA** | **NDI** | **ODI** |
| --- | --- | --- | --- | --- | --- | --- |
| Tract | **** | **** | **** | **** | **** | **** |
| Hemisphere | ns | * | ns | *** | ns | **** |
| Group | **** | *** | **** | ns | *** | **** |
| Tract : Hemisphere | **** | * | **** | **** | **** | **** |
| Tract : Group | **** | **** | **** | **** | **** | **** |
| Hemisphere : Group | ns | ns | ns | ns | ns | ns |

**B.**

|  | **PT_EV_ *vs* FT_EVCt_**  (N= 33) | | | **PT_ML_ *vs* FT_MLCt_**  (N = 26) | | |
| --- | --- | --- | --- | --- | --- | --- |
|  | **T** | **CI95%** | ***p*-value** | **T** | **CI95%** | ***p*-value** |
| **AD** | 22.49 | [0.; 0.] | **** | 6.02 | [0.;0.] | **** |
| **RD** | 20.98 | [0.; 0.] | **** | 5.49 | [0.;0.] | **** |
| **MD** | 22.73 | [0.; 0.] | **** | 5.87 | [0.;0.] | **** |
| **FA** | -11.47 | [-0.02;-0.01] | **** | -2.11 | [-0.01;0.] | ** |
| **NDI** | -21.76 | [-0.03;-0.02] | **** | -3.78 | [-0.01;0.] | *** |
| **ODI** | -13.84 | [-0.02;-0.01] | **** | -4.87 | [-0.01;0.] | **** |

**Table UniT2**. **Group comparisons per tract**: paired t-tests for each diffusion metric between PT_EV_ vs FT_EVCt_ (N=33 in each group). Left/Right tracts values are averaged, as for all the further supplementary tables. P-values are corrected for multiple comparisons.

|  | **AD** | | **RD** | | **MD** | | **FA** | | **NDI** | | **ODI** | |
| --- | --- | --- | --- | --- | --- | --- | --- | --- | --- | --- | --- | --- |
| **Tracts** | **T** | ***p*** | **T** | ***p*** | **T** | ***p*** | **T** | ***p*** | **T** | ***p*** | **T** | ***p*** |
| S1-Bstem | 5.39 | **** | 3.9 | *** | 5.01 | **** | -0.6 | ns | -3.28 | ** | -4.87 | **** |
| M1-Bstem | 5.0 | **** | 4.78 | **** | 5.42 | **** | -1.92 | . | -4.22 | *** | -3.09 | ** |
| ParaC-Bstem | 4.55 | *** | 6.11 | **** | 6.22 | **** | -3.53 | ** | -5.89 | **** | -1.83 | . |
| S1-Thal | 7.48 | **** | 5.6 | **** | 6.34 | **** | -2.6 | * | -6.22 | **** | -6.31 | **** |
| M1-Thal | 6.32 | **** | 4.7 | **** | 5.36 | **** | -1.88 | . | -5.34 | **** | -5.01 | **** |
| ParaC-Thal | 4.58 | **** | 4.94 | **** | 4.97 | **** | -3.55 | ** | -5.3 | **** | -2.91 | ** |
| S1-Caud | 8.12 | **** | 4.5 | *** | 5.76 | **** | -0.24 | ns | -5.42 | **** | -5.36 | **** |
| M1-Caud | 5.47 | **** | 2.93 | ** | 3.7 | ** | 0.08 | ns | -3.78 | *** | -6.16 | **** |
| ParaC-Caud | 5.23 | **** | 5.94 | **** | 6.05 | **** | -3.67 | ** | -6.35 | **** | -3.31 | ** |
| S1-Lenti | 6.84 | **** | 6.18 | **** | 6.52 | **** | -4.6 | **** | -6.15 | **** | -6.12 | **** |
| M1-Lenti | 5.62 | **** | 5.76 | **** | 5.91 | **** | -4.1 | *** | -5.98 | **** | -3.36 | ** |
| ParaC-Lenti | 6.11 | **** | 6.04 | **** | 6.33 | **** | -4.03 | *** | -6.47 | **** | -2.57 | * |
| S1_L_-S1_R_ | 5.75 | **** | 9.23 | **** | 9.08 | **** | -6.73 | **** | -7.86 | **** | -0.91 | ns |
| M1_L_-M1_R_ | 5.26 | **** | 8.23 | **** | 7.99 | **** | -6.39 | **** | -7.22 | **** | -3.64 | ** |
| S1-M1 | 6.94 | **** | 7.42 | **** | 7.4 | **** | -5.49 | **** | -7.24 | **** | -3.64 | ** |

Positive T values refer to higher values in FT than PT. Refer to *Table 1* for *p*-value legend and to *Figure 1* for metrics and tracts abbreviations.

2) In a second step, we aimed to evaluate whether clinical factors important for preterm development should be considered as confounders in the analyses of tract microstructure. We studied the effect of selected clinical factors (focusing on the ones previously reported as having significant effect on infant WM microstructure, cf. section ***Neonatal characteristics at birth***) on the diffusion metrics considering only the preterm infants, by performing additional ANOVA modelling considering all tracts (including interhemispheric tracts (**Table UniT3**). Confirming previous results, the tract effect was highly significant for all metrics. The group effect (PT_EV_ vs PT_ML_) as well as the interaction between the tract and group, were less significant than in the previous analyses, probably because only PT infants were considered while larger differences are observed between PT and FT than between PT_EV_ and PT_ML_. Globally, multiple pregnancy, IUGR and parenteral nutrition were weakly associated with some of the evaluated diffusion metrics. Interestingly, sex and preterm morbidities were not significantly associated with the diffusion metrics, which led us not to retain this latter variable in the next analyses. As a consequence, only the significant clinical factors were considered in further analyses.

**Table UniT3.** ANOVA analysis including only the preterm infants (PT_EV_ and PT_ML_ groups) to study the effect of selected clinical factors on each diffusion metric.

|  | **AD** | **RD** | **MD** | **FA** | **NDI** | **ODI** |
| --- | --- | --- | --- | --- | --- | --- |
| Tract | **** | **** | **** | **** | **** | **** |
| Group | * | ns | . | ns | . | * |
| Sex | ns | ns | ns | ns | ns | ns |
| Multiple Pregnancy | ns | ns | ns | ns | ns | * |
| IUGR | * | . | * | ns | * | * |
| Preterm Morbidities | ns | ns | ns | ns | ns | ns |
| Parenteral Nutrition >21d | * | . | . | ns | * | * |
| Tract : Group | ns | ** | * | **** | ns | ns |
| Group : Sex | ns | ns | ns | ns | ns | ns |
| Group : Multiple Pregnancy | ns | ns | ns | ns | ns | * |
| Group : IUGR | ns | ns | ns | ns | ns | * |
| Group : Preterm Morbidities | ns | ns | ns | ns | ns | ns |
| Group : Parenteral Nutrition | NA | NA | NA | NA | NA | NA |
| Sex : Multiple Pregnancy | ns | ns | ns | ns | ns | ns |
| Sex : IUGR | ns | ns | ns | ns | ns | ns |
| Sex : Preterm Morbidities | ns | ns | ns | ns | ns | ns |
| Sex : Parenteral Nutrition | ns | ns | ns | ns | ns | ns |

Refer to *Table 1* legend for abbreviations and *p*-values significance.

3) Our third aim was to evaluate the effects of additional continuous variables that are known to impact the tract microstructure: PMA at scan and whole-brain WM microstructure. GA at birth was then preferred to group categorization in order to better describe the inter-individual variability. Thus, we used a global ANCOVA model with PMA at scan, GA at birth and a proxy of whole-brain WM microstructure. For this latter variable (hereafter named WM residuals), we considered the residuals of the linear model considering the metric averaged over the whole WM mask as a function of GA at birth and PMA at scan, which were shown to be highly associated with the averaged WM metric (**Table UniT4. A**). In the model, we also considered factors that were previously relevant, among which the tracts and a few clinical factors.

For each metric, the modelling uncovered significant associations with the three continuous variables (PMA at scan, GA at birth and a proxy of whole-brain WM microstructure), but the effects of clinical factors, other than IUGR, dropped considerably in significance compared to the previous models probably because these factors are related to GA at birth and WM residuals, leading us not to retain them for the next multivariate analyses. Moreover, as the effect of IUGR was negligible compared to the effects of GA at birth, PMA at scan and WM residuals, this factor was likewise not retained in the subsequent analyses.

To further explore changes in diffusion metrics with GA at birth, we performed a linear regression over the whole cohort after correction for significant variables identified in the previous analyses (PMA at scan and WM residuals) (**Table UniT4. B**). Significant associations with GA at birth were observed in almost all SM tracts and metrics (**Table UniT4.B, Table UniT5** and **Figure UniF1**). Confirming the observations from the group analyses (**Figure 3, Table UniT2**), MD, AD, and RD metrics decreased, and FA, NDI, and ODI increased with the GA at birth. The lower associations for FA (only 10/15 tracts) and ODI (13/15 tracts) (**Table UniT5**) suggested that these metrics might be less sensitive to detect the variation of microstructure characteristics with GA at birth within some SM tracts, at least at TEA.

**Tables UniT4. A.** ANCOVA analysis including GA at birth (rather than the group belonging), PMA at scan, WM residuals (corrected for GA at birth and PMA at scan), relevant clinical factors (multiple pregnancy, IUGR, parenteral nutrition >21days), as well as relevant interactions between factors. **B.** Multiple linear regression over the whole cohort of mean WM metrics with PMA at scan and GA at birth, and interactions.

Refer to *Table 1* legend for abbreviations and *p*-values significance.

**A.**

|  | **AD** | **RD** | **MD** | **FA** | **NDI** | **ODI** |
| --- | --- | --- | --- | --- | --- | --- |
| Tract | **** | **** | **** | **** | **** | **** |
| GA at birth | **** | **** | **** | **** | **** | **** |
| PMA at scan | **** | **** | **** | **** | **** | . |
| Residual WM | **** | **** | ns | * | **** | ns |
| Multiple Pregnancy | * | ns | ** | . | ** | ** |
| IUGR | ns | ns | . | ns | * | ns |
| Parenteral Nutrition | ns | ns | * | . | *** | . |
| Tract : GA at birth | **** | **** | **** | **** | **** | **** |
| Tract : PMA at scan | ** | **** | **** | **** | **** | ns |
| Tract : Residual WM | **** | **** | . | **** | ** | *** |
| Tract : Multiple Pregnancy | ns | . | ** | ** | *** | ns |
| Tract : IUGR | ns | ns | ns | ns | ns | ns |
| Tract : Parenteral Nutrition | . | ** | *** | ns | *** | ns |

**B.**

|  | **AD** | **RD** | **MD** | **FA** | **NDI** | **ODI** |
| --- | --- | --- | --- | --- | --- | --- |
| PMA at scan | **** | **** | **** | **** | **** | **** |
| GA at birth | **** | **** | **** | **** | **** | * |
| PMA : GA at birth | ns | ns | ns | ns | ns | ns |

**Table UniT5.** Univariate linear regression analysis: relationship between SM tracts’ diffusion metrics (corrected for PMA at scan and WM residuals) and GA at birth over the whole cohort.

|  | **AD** | | **RD** | | **MD** | | **FA** | | **NDI** | | **ODI** | |
| --- | --- | --- | --- | --- | --- | --- | --- | --- | --- | --- | --- | --- |
| **Tracts** | T | *p* | T | *p* | T | *p* | T | *p* | T | *p* | T | *p* |
| S1-Bstem | -7.86 | **** | -4.83 | **** | -7.32 | **** | -0.33 | ns | 2.98 | ** | 6.28 | **** |
| M1-Bstem | -6.43 | **** | -5.22 | **** | -6.84 | **** | 0.69 | ns | 3.41 | ** | 3.74 | *** |
| ParaC-Bstem | -6.74 | **** | -7.58 | **** | -8.88 | **** | 2.89 | ** | 5.57 | **** | 1.79 | ns |
| S1-Thal | -9.88 | **** | -8.72 | **** | -9.81 | **** | 3.14 | ** | 8.29 | **** | 7.74 | **** |
| M1-Thal | -7.7 | **** | -7.22 | **** | -8.19 | **** | 1.89 | ns | 7.14 | **** | 5.24 | **** |
| ParaC-Thal | -5.76 | **** | -8.05 | **** | -7.87 | **** | 4.89 | *** | 7.53 | **** | 2.26 | * |
| S1-Caud | -11.1 | **** | -6.1 | **** | -8.19 | **** | 0.36 | ns | 6.82 | **** | 6.45 | **** |
| M1-Caud | -7.82 | **** | -3.04 | ** | -4.41 | **** | -1.19 | ns | 4.4 | **** | 7.3 | **** |
| ParaC-Caud | -6.42 | **** | -9.52 | **** | -9.21 | **** | 5.49 | *** | 9.34 | **** | 3.28 | ** |
| S1-Lenti | -10.2 | **** | -10.8 | **** | -11.7 | **** | 5.1 | *** | 10.2 | **** | 9.3 | **** |
| M1-Lenti | -7.87 | **** | -9.96 | **** | -10.1 | **** | 5.09 | *** | 9.49 | **** | 4.35 | **** |
| ParaC-Lenti | -8.2 | **** | -10.21 | **** | -10.9 | **** | 4.49 | *** | 9.26 | **** | 3.09 | ** |
| S1L-S1R | -7.22 | **** | -12.1 | **** | -12.6 | **** | 7.07 | *** | 9.16 | **** | 1.35 | ns |
| M1L-M1R | -6.62 | **** | -12.58 | **** | -11.8 | **** | 9.07 | *** | 10.58 | **** | 4.08 | *** |
| S1-M1 | -11.5 | **** | -15.68 | **** | -14.7 | **** | 9.41 | *** | 13.04 | **** | 4.93 | **** |

Refer to *Figure 1* and *Table 1* legends for abbreviations. P-values are corrected for multiple comparisons.

| 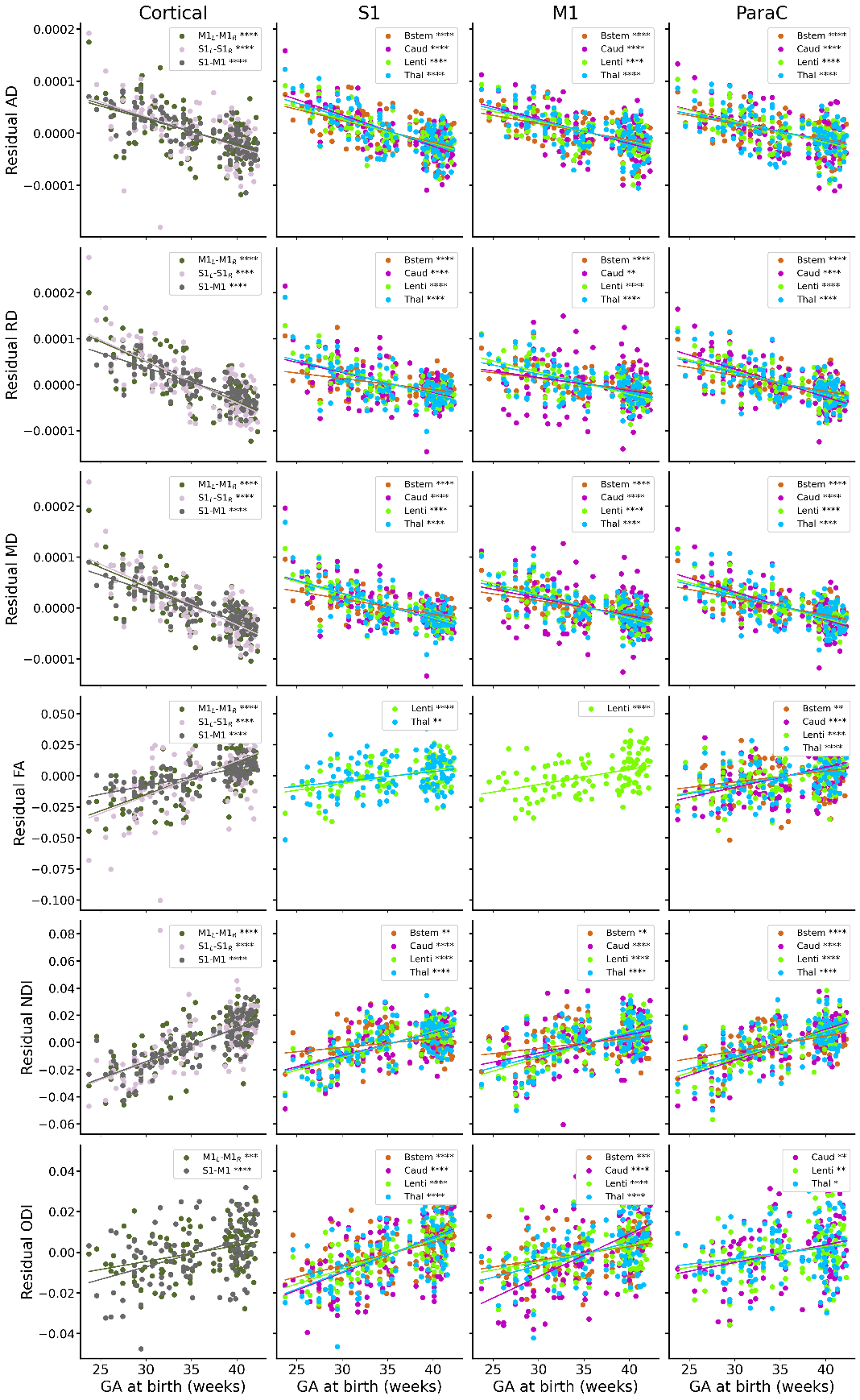 | 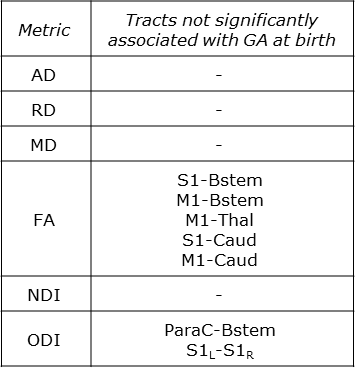 |
| --- | --- |

**Figure UniF1. Scatterplot of metrics residuals (after correction of PMA at scan and WM residuals) with GA at birth**. The regression lines show significant relationship with GA at birth (after correction for multiple comparisons). The table lists the tracts that are not significantly associated with GA at birth for each metric. Refer to *Figure 1* legend for abbreviations.

## *Results for BSID-III composite scores (CS)*

Composite scores below 85 (corresponding to <-1SD, indicating a developmental delay) concerned 16.5% of infants for Communication (N=16, 9 PT), 6.2% for Cognition (N=6, 4 PT) and 5.2% for Motricity (N=5, 3 PT).

**Table Sup-CS_T1**. Comparison of BSID-III composite scores at around 18 months of corrected age between PT and FT infants. p-values were obtained from paired t-tests corrected for multiple comparisons. See *Figure Sup-CS-F1* for distributions and graphical comparison between groups.

|  | **Preterm group**  (*n*=59) | | **Full-term controls**  (*n*=59) | |  |
| --- | --- | --- | --- | --- | --- |
| **Bayley’s scores** (BSID-III) | *NA* | Mean (SD)  [range] | *NA* | Mean (SD)  [range] | *p* |
| ***Composite* scores** | *15* |  | *6* |  |  |
| Cognitive |  | 100.7 (13.1) [55.0;125.0] |  | 100.8 (9.8) [70.0;125.0] | ns |
| Communication |  | 98.4 (18.3) [47.0;141.0] |  | 101.4 (15.5) [68.0;127.0] | ns |
| Motor |  | 100.4 (10.5) [70.0;124.0] |  | 101.6 (8.5) [73.0;118.0] | ns |


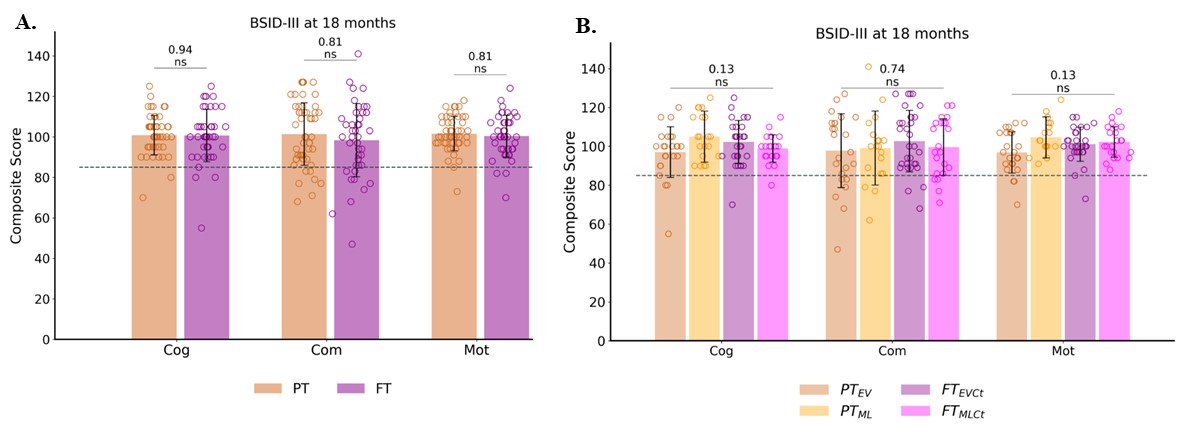


**Figure Sup-CS_F1.**Outcome assessment at around 18 months of corrected age: BSID-III composite scores between PT and FT groups (**A**), and subgroups (**B**: PT_EV_, PT_ML_, FT_EVCt_ and FT_MLCt_).

The dotted line indicates the scores threshold indicating a developmental delay (scores < -1 SD): composite scores < 85. Reported statistics are results of either t-test corrected for multiple comparisons for PT vs FT or one-way ANOVA for the subgroup analysis. Cog: cognitive; Com: communication; Mot: motor. ns: not significant.

**Table Sup-CS_T2.** Correlation analyses between Mahalanobis distance and BSID-III composite scores**.** Only PT_EV_ results for *set 3* (NDI and ODI) are shown (p-values corrected for multiple comparisons), as no significant correlations were found in *sets 1* and *2.*

|  | **PT_EV_ - Set 3 (NDI, ODI)** | | | | | |
| --- | --- | --- | --- | --- | --- | --- |
|  | **Cognitive** | | **Communication** | | **Motor** | |
| **Tract** | r | *p* | r | *p* | r | *p* |
| S1-Brainstem | -0.51 | ns | -0.41 | ns | -0.37 | ns |
| **M1-Brainstem** | -0.7 | * | -0.55 | ns | -0.49 | ns |
| **ParaC-Brainstem** | -0.66 | * | -0.48 | ns | -0.52 | ns |
| S1-Thal | -0.35 | ns | -0.19 | ns | -0.18 | ns |
| M1-Thal | -0.5 | ns | -0.4 | ns | -0.4 | ns |
| ParaC-Thal | -0.34 | ns | -0.3 | ns | -0.35 | ns |
| S1-Caud | -0.41 | ns | -0.06 | ns | -0.16 | ns |
| M1-Caud | -0.27 | ns | -0.14 | ns | -0.18 | ns |
| ParacC-Caud | -0.35 | ns | -0.38 | ns | -0.33 | ns |
| S1-Lenti | -0.52 | ns | -0.45 | ns | -0.36 | ns |
| M1-Lenti | -0.46 | ns | -0.49 | ns | -0.47 | ns |
| ParaC-Lenti | -0.45 | ns | -0.47 | ns | -0.49 | ns |
| S1L-S1R | -0.01 | ns | 0.03 | ns | 0.04 | ns |
| M1L-M1R | -0.47 | ns | -0.49 | ns | -0.45 | ns |
| S1-M1 | -0.56 | ns | -0.47 | ns | -0.48 | ns |

Refer to *Table 1* for p-value legend and to *Figure 1* for tracts abbreviations.
